# Dodecapeptide Cathelicidins of Cetartiodactyla: Structure, Mechanism of Antimicrobial Action, and Synergistic Interaction With Other Cathelicidins

**DOI:** 10.3389/fmicb.2021.725526

**Published:** 2021-08-13

**Authors:** Ilia A. Bolosov, Pavel V. Panteleev, Sergei V. Sychev, Stanislav V. Sukhanov, Pavel A. Mironov, Mikhail Yu. Myshkin, Zakhar O. Shenkarev, Tatiana V. Ovchinnikova

**Affiliations:** ^1^M. M. Shemyakin and Yu. A. Ovchinnikov Institute of Bioorganic Chemistry, Russian Academy of Sciences, Moscow, Russia; ^2^Phystech School of Biological and Medical Physics, Moscow Institute of Physics and Technology (State University), Dolgoprudny, Russia; ^3^Faculty of Biology, Lomonosov Moscow State University, Moscow, Russia

**Keywords:** antimicrobial peptide, cathelicidin, dodecapeptide, goat, synergy, NMR, bactenecin

## Abstract

In this study, dodecapeptide cathelicidins were shown to be widespread antimicrobial peptides among the *Cetruminantia* clade. In particular, we investigated the dodecapeptide from the domestic goat *Capra hircus*, designated as ChDode and its unique ortholog from the sperm whale *Physeter catodon* (PcDode). ChDode contains two cysteine residues, while PcDode consists of two dodecapeptide building blocks and contains four cysteine residues. The recombinant analogs of the peptides were obtained by heterologous expression in *Escherichia coli* cells. The structures of the peptides were studied by circular dichroism (CD), FTIR, and NMR spectroscopy. It was demonstrated that PcDode adopts a β-hairpin structure in water and resembles β-hairpin antimicrobial peptides, while ChDode forms a β-structural antiparallel covalent dimer, stabilized by two intermonomer disulfide bonds. Both peptides reveal a significant right-handed twist about 200 degrees per 8 residues. In DPC micelles ChDode forms flat β-structural tetramers by antiparallel non-covalent association of the dimers. The tetramers incorporate into the micelles in transmembrane orientation. Incorporation into the micelles and dimerization significantly diminished the amplitude of backbone motions of ChDode at the picosecond-nanosecond timescale. When interacting with negatively charged membranes containing phosphatidylethanolamine (PE) and phosphatidylglycerol (PG), the ChDode peptide adopted similar oligomeric structure and was capable to form ion-conducting pores without membrane lysis. Despite modest antibacterial activity of ChDode, a considerable synergistic effect of this peptide in combination with another goat cathelicidin – the α-helical peptide ChMAP-28 was observed. This effect is based on an increase in permeability of bacterial membranes. In turn, this mechanism can lead to an increase in the efficiency of the combined action of the synergistic pair ChMAP-28 with the Pro-rich peptide mini-ChBac7.5Nα targeting the bacterial ribosome.

## Introduction

Rapid growth of antimicrobial resistance along with challenges of novel conventional antibacterial agent discovery revealed the necessity to develop new approaches to combat infections (Czaplewski et al., 2016). Among them, host defense antimicrobial peptides (AMPs) came into a sharp focus as possible alternatives to conventional antibiotics. The complex membrane-targeting mechanism of their antimicrobial action and the ability to rapidly kill pathogens as well as a plethora of other biological functions make them a perspective scaffold for drug design. AMPs are highly diverse molecules across and even within species. Many plant and animal genomes are encoding several distinct AMP gene families (Lazzaro et al., 2020). As a result, host organisms naturally deploy them in synergistic cocktails that limit the probability of bacterial resistance evolution in nature.

Cathelicidins, one of the major and structurally diverse groups of animal AMPs, are known to be the key molecular factors of innate immunity of most vertebrate species. Cathelicidins are found in many vertebrates: mammals, birds, fishes, amphibians, and a hagfish (Kościuczuk et al., 2012). Secondary structures of mature cathelicidins may include α-helices, β-structure, and extended linear regions enriched with Trp or Pro residues. The number of cathelicidin genes varies greatly in different species, from the only one in humans (LL-37) to a dozen in artiodactyls. In the latter case, simultaneous expression of several cathelicidins can be observed. For example, it has been shown that three cathelicidins are most actively expressed in leukocytes of the goat *Capra hircus*: cathelicidin-1 (ChDode), cathelicidin-3 (ChBac7.5) and cathelicidin-6 (ChMAP-28) (Zhang et al., 2014).

**FIGURE 1:**
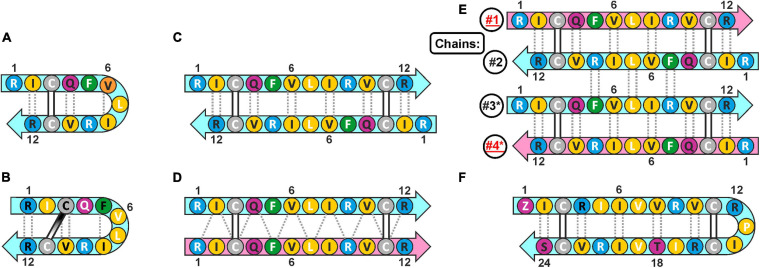
The possible secondary structures of monomeric and dimeric cathelicidins-1 (bactenecins) by the example of ChDode. **(A)** Monomeric β-hairpin with γ-turn proposed by computer modeling (Romeo et al., 1988). **(B)** Monomeric β-hairpin with β-turn according to NMR data measured in water (Raj et al., 2000). **(C)** Antiparallel disulfide linked homodimer according to NMR data measured in water (present report). **(D)** Proposed structure of parallel disulfide linked homodimer (Storici et al., 1996). **(E)** Antiparallel non-covalent dimer of the antiparallel disulfide linked homodimers according to NMR data measured in DPC micelles (present report). The chains of the tetramer are numbered. The * marks the chains of the second homodimer in the tetramer. **(F)** Monomeric 24-residue β-hairpin cathelicidin-1 from the sperm whale *Physeter catodon* (present report). The residues of the peptide are color coded according to their properties. The aromatic, hydrophobic, basic, polar, and Cys residues are shown as green, yellow, blue, magenta, and gray circles, respectively. The residues with side-chains directed up from the picture plane (toward readers) are marked by black labels; the residues with side chains directed down from the picture plane are marked by white labels. The mainchain–mainchain hydrogen bonds are shown by dotted lines. In asymmetric oligomers **(D,E)** the non-symmetrical β-strands (peptide units) are colored differently.

Cathelicidins-1 (also known as bactenecins or dodecapeptides) are small antimicrobial peptides, first isolated from the neutrophilic granulocytes of representatives of the *Bovidae* family. Catelicidin-1 precursor proteins are encoded by the *CATHL1* genes, and consist of the *N*-terminal conserved cathelin-like domain (CLD), an elastase processing site, and a *C*-terminal mature peptides part. In general, mature peptide consist of 12 amino acid residues, and have two cysteine residues at positions 3 and 11 (Romeo et al., 1988). The bovine bactenecin exhibits antibacterial and antibiofilm activity, though data on its structure and mechanism of action are quite contradictory (Panteleev et al., 2017). According to early reports, two Cys residues form intramolecular disulfide bond resulting in a 9-membered ring (Romeo et al., 1988). The cyclic peptide adopts a β-hairpin conformation with γ- (Figure 1A; Romeo et al., 1988) or β-turn (Figure 1B; Raj et al., 2000) on its tip. A number of monomeric bactenecin analogs that have an increased therapeutic index have been obtained. For example, the analog IDR-1018, which has broad-spectrum activity (antimicrobial, anti-inflammatory, wound-healing, and others), is considered as a new generation immunomodulator (Mansour et al., 2015), and the analog IMX942 is currently undergoing the second phase of clinical trials as an antibiotic for patients with febrile neutropenia (Fjell et al., 2011). However, the later data argue that a native form of the peptide can be an antiparallel or parallel β-structural dimer (Figures 1C,D), where Cys residues form two intermolecular disulfide bridges (Storici et al., 1996).

Here we report recombinant production and structure-function study of cathelicidin-1 from *C. hircus*. The natural variability of *CATHL1* genes in Cetartiodactyla species was also analyzed. According to circular dichroism (CD), Fourier transform infrared (FTIR) and NMR spectroscopy data, ChDode in water has the structure of covalent highly twisted antiparallel β-structural homodimer (24 residues), which undergo further oligomerization with formation of a large flat β-sheet in membrane-mimicking environment. The structure of the symmetric antiparallel dimer of the covalent ChDode dimers (Figure 1E) was determined by NMR spectroscopy in the environment of dodecyl-phosphocholine (DPC) micelles. We showed that ChDode possessed membrane-permeabilizing activity and formed toroidal pores when exposed to a model membrane containing anionic lipids. An indirect confirmation of the homodimeric arrangement of the Cetartiodactyla cathelicidins-1 was obtained from analysis of the sperm whale *Physeter catodon* cathelicidin-1 (PcDode). This peptide consists of two fused dodecapeptide building blocks and contains four cysteine residues. According to results of NMR analysis PcDode adopts β-hairpin structure (24 residues) stabilized by two intramolecular disulfide bonds.

Earlier we have shown the synergistic antibacterial effects between two goat cathelicidins: the membrane active ChMAP-28 and the truncated form of ChBac7.5 (mini-ChBac7.5Nα) targeting the bacterial ribosome (Panteleev et al., 2018). Therefore, here we studied the biological activity of ChDode and its ability to increase permeabilization of outer and cytoplasmic membrane of *Escherichia coli* individually and in combination with ChMAP-28 and mini-ChBac7.5Nα. The role of each of the three major goat cathelicidins in synergistic interaction was elucidated.

## Materials and Methods

### Identification of CATHL1 Genes in Cetartiodactyla WGS Database

At the first stage, the TBLASTN program was used to identify *CATHL1* genes in whole-genome shotgun (WGS, GenBank) database using conservative cathelin-like domain (CLD) fragment FRVKETVCPRTTQQPPEQCDFKE encoded by nucleotide sequence located in the second exon of the goat cathelicidin-1 (GenBank: XM_018038479.1) as a query sequence using the values of the default parameters (matrix: BLOSUM62, gap costs: existence 11, extension 1). Then, the obtained hit DNA contigs (±1,500 bp relative to the query) were analyzed by GenScan program^1^ to identify exons within genomic sequence. The putative elastase processing sites in fourth exon were suggested based on information about known Cetartiodactyla cathelicidins. Finally, putative mature cathelicidin sequences were manually (visually) inspected and additionally analyzed by CAMP database instruments^2^ to identify CATHL1-like 12-residue peptides containing two cysteine residues.

### Recombinant Production of the Cathelicidins-1

The ChDode (RICQFVLIRVCR) and PcDode (QICRIIVVRVCRPICRITVIRVCS) primary structures were deduced from the mRNA sequences (GenBank: XM_018038479.1 and XM_007124827.2) of the corresponding precursor proteins. Oligonucleotides coding whole 12-residue peptide (forward primer: 5′-GCA GAT CTA TGC GTA TCT GTC AGT TTG TTT TAA TTC GCG TGT GTC GTT AAG AAT TCG C-3′; reverse primer: 5′-GCG AAT TCT TAA CGA CAC ACG CGA ATT AAA ACA AAC TGA CAG ATA CGC ATA GAT CTG C-3′) and 24-residue peptide (forward primer: 5′-GCA GAT CTA TGC AGA TTT GCC GCA TTA TTG TGG TGC GTG TAT GTC GCC CAA TCT GTC-3′; reverse primer: 5′- GCG AAT TCT TAG CTG CAA ACA CGA ATA ACT GTA ATG CGA CAG ATT GGG CGA CAT ACA -3′) were designed on the basis of *E. coli* codon usage bias. The fragments coding target cathelicidins were generated by annealing of two primers. The fragments were then cloned into pET-based expression vectors as described previously (Panteleev et al., 2016). The target peptides were expressed in *E. coli* BL21 (DE3) as fusion proteins that included His-tag, the *E. coli* thioredoxin A [M37L], methionine residue, and a mature cathelicidin. ^15^N-labeled as well as unlabeled cathelicidins-1 were expressed in bacterial cells in M9 minimal medium containing 1 g/L ^15^NH_4_Cl (or NH_4_Cl, respectively), 20 mM glucose, 100 mg/L ampicillin, 1 mM MgSO_4_, and trace metals mixture. The cells were grown up to OD_600_ 1.0 and then were induced with 0.2 mM isopropyl β-D-1-thiogalactopyranoside (IPTG). The protein expression was performed at 30°C for 16 h with a shaking speed of 220 rpm. Then the cells were pelleted by centrifugation and sonicated in immobilized metal affinity chromatography (IMAC) loading buffer containing 6 M guanidine hydrochloride. The clarified lysate was applied on a column packed with Ni Sepharose (GE Healthcare). The recombinant protein was eluted with the buffer containing 0.5 M imidazole. Then the eluate containing the fusion protein was acidified (up to pH 1.0) and cleaved by 100-fold molar excess of cyanogen bromide over methionine for 20 h at 25°C in the dark. The reaction products were lyophilized, dissolved in water, titrated to pH 5.0, and loaded on a semi-preparative Reprosil-pur C18-AQ column (10 mm × 250 mm, 5-mm particle size, Dr. Maisch GmbH). Reversed-phase high-performance liquid chromatography (RP-HPLC) was performed with a linear gradient of acetonitrile (0–80% for 65 min) in water containing 0.1% trifluoroacetic acid with a flow rate of 2 ml/min. The peaks were monitored at 214 and 280 nm. The collected fractions were analyzed by MALDI-TOF mass-spectrometry using Ultraflex instrument (Bruker Daltonics) (see Supplementary Material). For PcDode, the *N*-terminal glutamine was additionally modified to form pyroglutamic acid. The peptide was dissolved in 0.2% TFA at a concentration of 1 mg/ml followed by incubation for 24 h at 37°C. In this conditions, spontaneous cyclization of glutamine occurred (Schilling et al., 2008). The modified PcDode was then purified by RP-HPLC. The recombinant goat cathelicidins ChMAP-28 and mini-ChBac7.5Nα were obtained as described previously (Panteleev et al., 2018).

### Antimicrobial Assay

Antimicrobial assay against Gram-negative bacteria (*E. coli* strain ML-35p) and Gram-positive bacteria (*Staphylococcus aureus* strain 209P) was performed as described previously (Wiegand et al., 2008; Panteleev et al., 2018). To verify MIC values the respiratory activity of the bacteria was determined. Briefly, 20 μl of 0.1 mg/ml resazurin (a redox indicator, Sigma) was added to the wells after 24 h of incubation, and the plate was incubated for an additional 2 h. The reduction of resazurin to resorufin was measured. The results were expressed as the median values of three experiments performed in duplicate. In all experiment series, no significant divergence was observed (within ±1 dilution step). All concentrations and ratios given in the manuscript for ChDode refer to the covalent homodimer (3 kDa).

### Checkerboard Assay

Checkerboard assay was performed as described previously (Berditsch et al., 2015; Panteleev et al., 2018). Briefly, estimation of synergistic effects of different cathelicidins was performed by calculating the fractional inhibitory concentration index (FICI) according to the equation: FICI = [A]/MIC_*A*_ + [B]/MIC_*B*_, where MIC_*A*_ and MIC_*B*_ are the MICs of the individual substances, while [A] and [B] are the MICs of A and B when used together. A synergistic effect was defined at a FICI ≤ 0.5.

### Bacterial Membranes Permeability Assay

To examine an ability of the peptides to affect barrier function of outer and inner membranes of Gram-negative bacteria, a colorimetric assay with chromogenic markers nitrocefin (Calbiochem-Novabiochem) and *o*-nitrophenyl-b-D-galactopyranoside (ONPG, AppliChem) and *E. coli* ML-35p strain was performed as previously described (Shamova et al., 2009; Panteleev et al., 2018). Briefly, the final concentration of *E. coli* ML-35p cells was of 2.5 × 10^7^ CFU/ml. The concentrations of ONPG and nitrocefin were of 2.5 mM and 20 mM, respectively. Peptides were serially diluted in a 96-well plate with a non-binding surface (NBS, Corning #3641), and the optical density (OD) of the solution rising due to the appearance of the hydrolyzed nitrocefin or ONPG was measured at 492 and 405 nm, respectively the microplate reader AF2200 (Eppendorf). The assay was performed in phosphate buffered saline (PBS) at 37°C under stirring at 500 rpm. Control experiments were performed under the same conditions without the addition of a peptide. The optical absorption of the solution after incubation with melittin for 2 h was taken as 100%. The absorbance of control wells containing no peptides was subtracted from the absorbance value of each well. Three independent experiments were performed, and the curve patterns were similar for all three series.

### Cell-Free Protein Expression Assay

In order to investigate the effect of AMPs on the translation process, the peptides were added to a cell-free protein synthesis (CFPS) reaction mix with a plasmid encoding enhanced green fluorescent protein (EGFP) variant (F64L, S65T, Q80R, F99S, M153T, and V163A) under a control of the T7 promoter as described previously with some modifications (Panteleev et al., 2018). Briefly, the peptides were dissolved 0.1% BSA in water. Streptomycin was used in the positive control reactions. Fluorescence of the sample without inhibitor was set as the 100% value. The reaction proceeded for 2 h in 96-well flat-bottom black polystyrene microplates in a plate shaker (30°C, 900 rpm). Fluorescence of the synthesized EGFP was measured with the microplate reader AF2200 (λ_*Exc*_ = 488 nm, λ_*Em*_ = 510 nm). The experimental data were obtained from at least three independent experiments performed in duplicate. IC_50_ values were determined by interpolation from non-linear regression curves using the GraphPad Prism 6.0 software.

### Hemolysis and Cytotoxicity Assay

Hemolytic activity of the peptides was tested against the fresh suspension of human red blood cells (hRBC) using the hemoglobin release assay as described previously (Lee et al., 2007; Panteleev et al., 2016). Three experiments were performed with the hRBC from blood samples obtained from independent donors. The obtained data were represented as average means with standard deviations. The cytotoxicity of the peptides against human keratinocytes (HaCaT) cell lines was studied using the colorimetric 3-(4,5-dimethylthiazol-2-yl)-2,5-diphenyltetrazolium bromide (MTT) dye reduction assay according to Panteleev and Ovchinnikova (2017). Three independent experiments were performed for each peptide. Half maximal inhibitory concentration (IC_50_) values were estimated as described previously (Kuzmin et al., 2018).

### CD and FTIR Spectroscopy

The following lipids were used for preparation of small unilamellar vesicles (SUVs): dimyristoyl-phosphatidylethanolamine (DMPE) (Fluka, Buchs, Switzerland), soybean phosphatidylcholine (PC), (Avanti Polar Lipids, Alabaster, AL, United States), dimyristoyl-phosphatidylglycerol (DMPG) (Lipoid GmbH, Ludwigshafen, Germany). To prepare the ChDode-containing SUVs, the peptide (0.15–1.3 mg) was dissolved in ethanol (0.6 ml) and mixed with required amounts of lipids in chloroform (0.6 ml) to the final dimer-to-lipid molar ratio (D:L) of 1:120. Then solvents were removed in rotary evaporator at 45°C, and the samples were dried for 1 h under medium vacuum (∼10^–3^ Torr). The peptide-lipid film was dissolved in 10 mM sodium phosphate buffer (NaPi, pH 7.2) to the final ChDode concentration of 1.5 mM for FTIR, and 0.15 mM for CD measurements. The samples were incubated for 0.5 h at 20°C and then were sonicated on ice with a Q125 sonicator (Qsonica, Newtown, CT, United States) for 1.5 min until they became optically clear. The diameter of the obtained SUVs was determined by dynamic light scattering on a Coulter N4 MD particle analyzer (Hialeah, FL, United States). The measured diameter of liposomes was of 55 ± 10 nm. For FTIR measurements in the DPC micelles, the concentrated aqueous solution of DPC (Avanti Polar Lipids) was added to the peptide sample in NaPi (pH 7.2) to the final D:L of 1:130. CD measurements were done using ChDode/d38-DPC sample after NMR measurements (dimer concentration of 0.2 mM, D:L = 1:130, pH 4.0) (Sychev et al., 2017b).

Far-UV CD spectra were measured using a Jasco J-810 spectropolarimeter (Tokyo, Japan) at 25°C in demountable cells (Hellma, Mulheim/Baden, Germany) with 100 μm path length. Four scans were averaged. FTIR spectra were measured at 20°C on a Perkin-Elmer 1725 × Spectrometer (Beaconsfield, United Kingdom) with TGS detector and with hermetic interferometer area, which was sealed and fitted with two boxes of molecular sieves. The sieve boxes were baked at 250°C for 8 h before measurements. Buffer was deaerated *in vacuo* for 1 min. The cell thickness was determined from interference patterns of empty cells. The Perkin-Elmer cells with path lengths of 105 μm were used for the measurements in ethanol. 150 scans were averaged with a resolution of 4 cm^–1^. Because of strong water bending mode at ∼1,645 cm^–1^ FTIR spectra in NaPi buffer and in aqueous suspensions of liposomes or micelles were measured in very thin cuvettes (12 μm). 200 scans were averaged with a resolution of 4 cm^–1^. The spectra of liposomes or micelles without the peptide were subtracted. The curve analysis and fitting were carried out using the OriginPro 8.5 (OriginLab Corp., Northampton, MA, United States) software. The fragments of FTIR spectra were fitted to the sum of several Lorentz lines.

### NMR Spectroscopy

NMR study was performed using the samples containing 0.2–0.7 mM of non-labeled (ChDode or PcDode) or ^15^N-labeled (ChDode) peptide in 5% D_2_O at pH 4.0 (ChDode) or pH 4.4 (PcDode). For the NMR measurements in micellar solutions, the d38-DPC (CIL, Andover, MA) or lyso-myristoyl-phosphatidylglycerol (LMPG) (Avanti Polar Lipids) micelles were added to the aqueous ChDode samples using aliquots of a concentrated solutions until D:L ratio of 1:130 was reached. The NMR spectra were recorded on Bruker Avance III 600 and Bruker Avance III 800 spectrometers equipped with cryoprobes. The standard gradient enhanced pulse sequences (Rule and Hitchens, 2006) from Bruker Topspin library were used. Water suppression was achieved by ^15^N coherence selection by gradients (echo-antiecho) or by excitation sculpting. The spectra in water and in micelles solution were measured at 30°C and 40°C, respectively. ^1^H and ^13^C resonance assignments were obtained by a standard procedure based on a combination of 2D ^1^H-TOCSY, ^1^H-NOESY and natural abundance ^13^C-HSQC spectra using the CARA software. For ^15^N-labeled ChDode, the ^15^N resonance assignments and additional data were obtained using combination of 2D ^15^N-HSQC and 3D NOESY-^15^N-HSQC spectra.

Spatial structures were calculated using the CYANA 3.98 program (Schmidt and Güntert, 2015). Upper interproton distance constraints were derived from cross-peaks observed in 2D NOESY (τ_*m*_ = 100 ms) spectra via a “1/r^6^” calibration. For ChDode study in DPC micelles the data from 3D NOESY-^15^N-HSQC (τ_*m*_ = 150 ms) were also used. The ambiguous distance restraints were used during dimer of the dimers structure calculation (DPC micelles) in the cases where intramolecular contacts cannot be distinguished from the intermolecular ones. Torsion angle restraints and stereospecific assignments in water were obtained from ^3^J_*H*_^*N*^_*H*_^*A*^ and ^3^J_*H*_^*A*^_*H*_^*B*^ coupling constants estimated by line-shape analysis in 2D TOCSY spectra. The hydrogen bonding restraints were applied on the basis of temperature coefficients of amide protons measured in the range of 15–45°C. Additional symmetry-based angle restrains were introduced to keep the identity of the two chains in the covalent dimer of ChDode in water and the identity of inner chains of the tetramer and the identity of outer chains of the tetramer in DPC micelles.

Relaxation parameters (R_1_, R_2_, ^15^N-{^1^H}-NOE) of ^15^N nuclei were obtained using the standard set of ^15^N-HSQC based pseudo 3D experiments measured at 800 MHz. Analysis of relaxation data was performed in FastModelFree software (Cole, 2003) using isotropic rotational model. Hydrodynamic calculations were performed in the HYDRONMR software (García de la Torre et al., 2000). To probe the topology of the peptide/micelle complex the 1.5 mM GdCl_3_ and 3 mM DOTA chelating agent was added to the 0.2 mM ChDode sample in DPC micelles. The paramagnetic broadening of the HN resonances was qualitatively estimated using signal intensities in the 2D ^15^N-HSQC spectrum.

The overall geometry of the ChDode and PcDode β-sheets in the terms of ‘kink,’ ‘twist,’ and ‘interdimer cross’ (only for ChDode in DPC) angles was analyzed using method described in Stavrakoudis et al. (2009) (see Supplementary Material). Data in the NMR part of the manuscript are reported as mean ± S.D.

### Measurements of Light-Induced pH Changes in the Bacteriorhodopsin-Containing Proteoliposomes

Delipidated bacterioopsin was prepared from purple membranes of *Halobacterium halobium* as described in Bayley et al. (1982). Reconstitution of delipidated bacterioopsin and formation of bacteriorhodopsin-phospholipid proteoliposomes was carried out by cholate dialysis (Bayley et al., 1982). The protein to lipid molar ratio was 1:1700. All-trans retinal (Type XVI) was from Sigma. The diameter of proteoliposomes measured by Coulter N4 MD particle analyzer was 40–45 nm for DMPE-containing liposomes and 55–65 nm for the others.

To measure light-induced pH changes in the proteoliposomes, 200 μl of proteoliposome suspension (protein concentration 0.25 mg/ml) was added to 2 ml of 1M NaCl (if not overwise stated) so the lipid concentration in the cell was 2.5 mM. The peptide solution was added to the proteoliposomes with rapid stirring so that peptide-to-lipid molar ratio was ∼1:1000. The measurements were conducted in a thermostated cell at 25°C with rapid stirring. pH was monitored with Cole-Parmer RZ-05658-65 electrode (Beverly, MA, United States) carefully shielded from radiation by foil. For removal of membrane/protein deposits, the electrode was cleaned by electrode cleaning solution (Oakton Instruments, Vernon Hills, IL, United States). The samples were illuminated with 500-Watt halogen lamp (OSRAM) from 35 cm distance using <480 nm cut-off yellow filter (Sychev et al., 2015).

### Preparation of Planar Bilayers and Electrochemical Measurements

The BLM mimicking the plasma membrane of Gram-negative bacteria was made of polar lipid extract from *E. coli* (Avanti Polar Lipids) consisting of phosphatidylethanolamine (PE), phosphatidylglycerol (PG), and cardiolipin (DPG) in 67:23:10 ratio (weight percent). BLMs were formed from lipid solution in n-decane by Muller technique (Mueller et al., 1962) on 0.85 mm orifice in a Teflon partition separating two compartments of 2 ml each. ChDode was added to *cis* compartment to final concentration of 1.67 μM. The current was recorded under potential of 50 mV. The measurements were taken in 5 mM HEPES, 20 mM NaCl, pH 7.4, at 20°C. Membrane conductance (*g*) was calculated from Ohm’s law, *g* = *I/V*, in which *I* is current and *V* is voltage. Other experimental details are described in Andrä et al. (2009) and Shenkarev et al. (2011).

### Accessing Codes

Experimental restraints and atomic coordinates for the ChDode dimer in water and the tetramer in DPC micelles and PcDode monomer in water have been deposited in PDB^3^ under accession codes 7ACE, 7ACB, and 7OSC, respectively. Dates of deposition 2020-09-10, 2020-09-10, and 2021-06-08, respectively.

## Results

### CATHL1 Genes Analysis in the Cetartiodactyla Order

**FIGURE 2:**
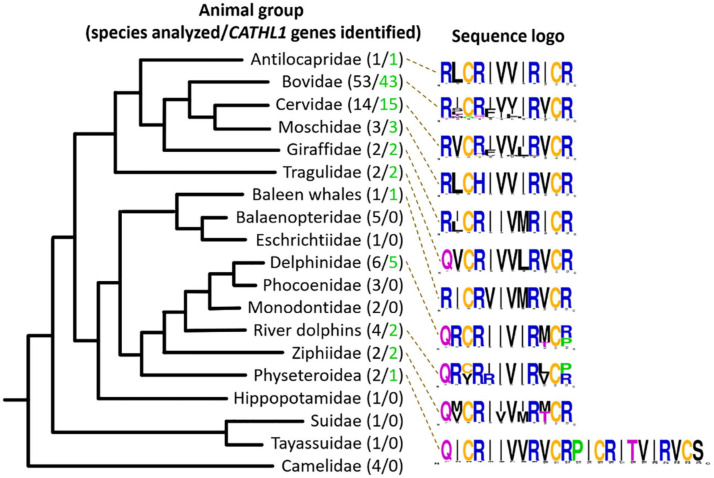
Analysis of CATHL1 genes orthologs from the Cetartiodactyla clade. Target genes were identified in the whole-genome shotgun (WGS, GenBank) database. The presented Cladogram was adapted from Beck et al. (2006). Amino acid frequency (sequence logo) graph was plotted using the WebLogo server (https://weblogo.berkeley.edu/).

In this study, 108 Cetartiodactyla species were analyzed and 77 target peptide sequences were identified (Figure 2). The obtained data suggest that dodecapeptide genes origin postdates disjoining representatives of the Cetruminantia clade and other ones of the Artiodactyla order, in particular, those of the Suidae family and the Tylopoda suborder. We did not precisely analyze *CATHL1* gene structures and its possible pseudogenization among the Cetartiodactyla order, however, stop codons in mature cathelicidin sequences were found in genomes of the Chinese muntjac *Muntiacus reevesi* and of the African buffalo *Syncerus caffer* (Supplementary Table 1). A significant variability of elastase processing site, which is present in the fourth exon of the known Cetartiodactyla *CATHL1* genes, was found as well. Notably, most of the identified genes encode a 12-residue mature peptide (dodecapeptide) except the 24-residue cathelicidin-1 from the sperm whale *Physeter catodon* (Figure 1F). This peptide contains 4 cysteine residues and consists of two dodecapeptide building blocks without a linker. The gene encoding this peptide as well as the *ChDode* gene displayed all the characteristics of a functional cathelicidin (Whelehan et al., 2014) including (1) the gene size of about 2 kb, (2) a conserved four exons/three introns arrangement with intact splicing sites, (3) a TATA-box just upstream from the transcription start site, (4) a polyadenylation signal located about 80 bp away from the stop codon (Supplementary Figure 1).

### Expression and Purification

Natural cathelicidins do not undergo significant post-translational modifications, therefore heterologous expression in *E. coli* of the peptides fused with a carrier protein seems to be a reasonable approach for their production. In this study, the modified thioredoxin A (TrxA) was used as a fusion partner that increased solubility of the recombinant protein, promoted the correct disulfide bond formation, and masked the toxic effects of AMPs (Parachin et al., 2012). A poor nutrient medium M9 was used for production of both native and ^15^N-labeled peptide. The fusion protein was expressed in *E. coli* BL21 (DE3) cells, and the obtained total cell lysates were fractionated by affinity chromatography. After purification and cleavage of the fusion protein, reverse-phase high performance liquid chromatography (RP-HPLC) was used for the fine purification of the mature recombinant peptide. Recombinant PcDode contains *N*-terminal glutamine residue. Usually, in natural peptides, it is modified into pyroglutamic acid (5-oxoproline) spontaneously or enzymatically by glutaminyl cyclases. To perform this modification *in vitro*, the purified PcDode was incubated overnight at 37°C and pH ∼ 2 as proposed in Schilling et al. (2008). The interconversion of *N*-terminal glutamine into cyclic form was confirmed by mass spectrometry and NMR spectroscopy (see below).

Final yields of both labeled and unlabeled ChDode as well as unlabeled PcDode were of about 5 mg per liter of the cell culture. Sodium dodecyl sulfate polyacrylamide gel electrophoresis (SDS-PAGE) showed that the cell lysates after induction contain the TrxA-ChDode fusion protein, synthesized predominately in the monomeric form, with a minor fraction of the dimeric form (Supplementary Figure 2). This can be explained by an influence of the *E. coli* cytoplasm redox potential. At the same time, after cell destruction, purification, and cleavage of the fusion protein the major part of ChDode peptide formed a covalent dimer. The experimental m/z values of 3007.22 and 2778.62 measured by MALDI-TOF mass spectrometry corresponded well to the doubled molecular mass of the 12-residue ChDode (3011.70 Da, [M + H]+) and monomeric 24-residue PcDode with *N*-terminal pyroglutamate (2782.56 Da, [M + H]+), and also the ∼4 Da difference in both cases due to formation of two intermolecular or intramolecular disulfide bonds.

The molecular mass of the ^15^N-labeled ChDode was increased by 44 Da indicating that all the ^14^N atoms were substituted with the stable isotopes ^15^N (Supplementary Figure 3). Interestingly, a small amount of the monomeric ChDode is visible in the MALDI-TOF spectrum.

### Secondary Structure of ChDode in Isotropic Solvents: CD and FTIR Spectroscopy

**FIGURE 3:**
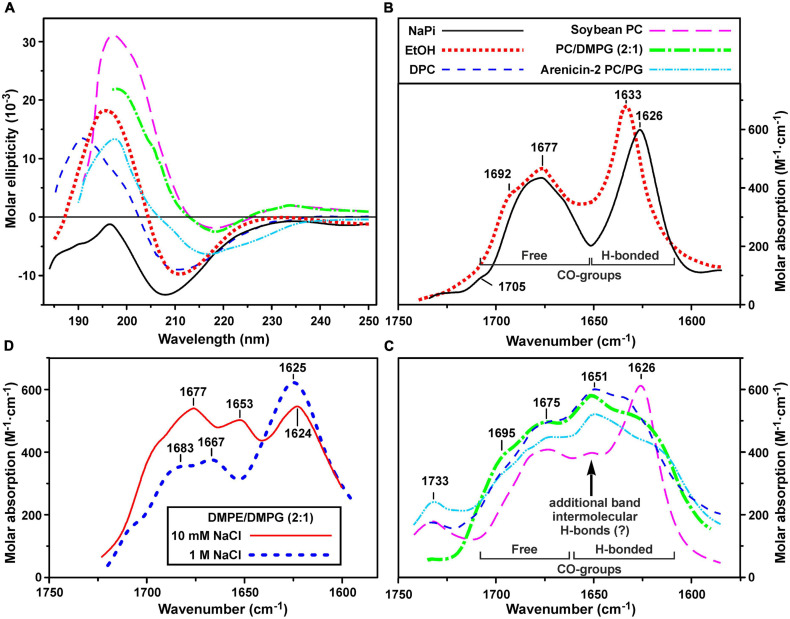
Circular dichroism (CD) spectra **(A)** and amide I region of FTIR spectra **(B–D)** of ChDode in different environments. **(A–C)** Spectra in 10 mM phosphate buffer (pH 7.2), ethanol, DPC micelles (pH 4.0), soybean phosphocholine (PC) small unilamellar vesicles (SUVs, 10 mM NaPi, pH 7.2), soybean PC/DMPG (2:1) SUVs (NaPi, pH 7.2) are shown. Please see the used designations for **(A–C)** above **(B)** panel. The spectra of arenicin-2 in PC/PG (7:3) SUVs (Sychev et al., 2017a,b) are shown on the panels **(A,C)** for comparison. On the panel **(C)**: the intensity of FTIR spectrum in PC SUVs is lowered for clarity. Weak band at 1,733 cm^– 1^ is due to incomplete compensation of the phospholipid ν(C = O) absorption. **(D)** FTIR spectra in DMPE/DMPG (2:1) SUVs (10 mM NaPi, pH 7.2) in the presence of 10 mM or 1 M NaCl. The intensity of the last spectrum is lowered for clarity.

Secondary structure of ChDode was analyzed in the different environments by CD and FTIR spectroscopy. CD spectra of the peptide in isotropic solvents – water (NaPi buffer, pH 7.2) and ethanol, are shown in Figure 3A. The spectrum in NaPi buffer showed two bands: strong minimum at 208 nm (amide *n* → π^∗^ transition) and weak maximum near 195 nm (amide π → π^∗^ transition). This spectrum resembled the spectra of parallel and antiparallel covalent dimers of bovine bactenecin in the same environment (Lee et al., 2008). The weak intensity of band at 195 nm indicated the presence of unordered or flexible regions in the peptide structure (Greenfield and Fasman, 1969). The ChDode spectrum in the less polar solvent, ethanol, with two strong bands at 210 nm and 196 nm was typical for an antiparallel (↑↓) or parallel (↑↑) β-structure and was similar to the spectrum of bovine bactenecin in SDS micelles (Lee et al., 2008). However, it should be noted that the CD spectra of small disulfide-rich β-structural peptides are often difficult to interpret due to the significant contribution of aromatic residues (Phe, Tyr, and Trp), disulfide bridges, and distortions (kinks and twists) of the β-structure (Chakrabartty et al., 1993).

To obtain an additional structural information, we analyzed the bands in the amide I region of FTIR spectra which are mainly associated with the C = O stretching vibration (Myshakina et al., 2008). Two major bands were observed in this spectral region in water and ethanol (Figure 3B). The first band at 1,626–1,633 cm^–1^ (with the weak shoulder at 1,692–1,705 cm^–1^) corresponds to the backbone C = O-groups participating in formation of hydrogen bonds (H-bonds) within the antiparallel β-structure. The second band at 1,677 cm^–1^ is attributed to free weakly solvated amide carbonyls which are not involved in intramolecular H-bonds. These assignments are based on the studies of model compounds (Hollósi et al., 1994; Jackson and Mantsch, 1995; Sychev et al., 2017b). The standard decomposition into Lorentzian lines gives the ratio of the integrated intensities of the bands of free and H-bonded C = O groups of 1.01 ± 0.05 and 1.13 ± 0.05 for ethanol and buffer, respectively, that is close to the value (∼1.0) expected for an antiparallel β-structure, where every second carbonyl forms a H-bond. Summarizing results obtained by CD and FTIR spectroscopy we can conclude, that ChDode in the isotropic solvents is an antiparallel β-structural dimer and the distortions in this structure (kink and/or twist) decrease with decreasing solvent polarity.

### NMR Structure of the ChDode Dimer in Aqueous Solution

The ChDode peptide contains only one acidic group – the *C*-terminal carboxyl. In proteins and peptides, the typical pKa value of *C*-terminal carboxyls is 3.3 ± 0.8 (Grimsley et al., 2009). Therefore, we used pH 4.0 for NMR study of ChDode. Under these conditions, the ionization state of the ChDode peptide should be close to the state at neutral pH 7.0 (total charge of the dimer –+6). The spatial structure of ChDode was studied using the unlabeled and ^15^N-labeled peptide variants.

**FIGURE 4:**
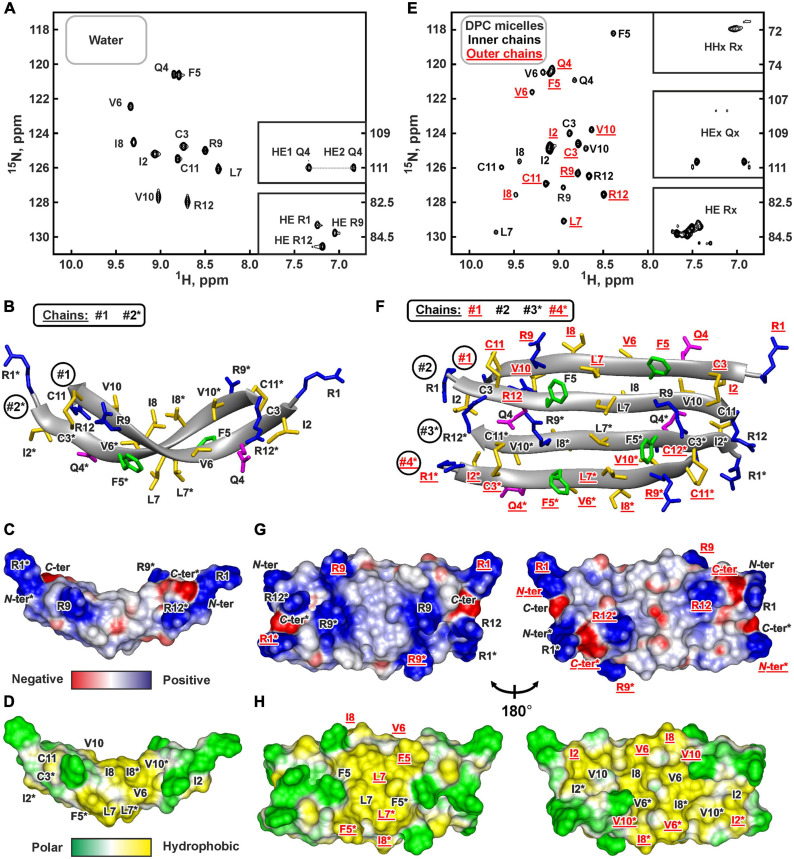
NMR data defines 3D structure of the ChDode dimer in water **(A–D)** and of the ChDode tetramer in DPC micelles **(E–H)**. **(A,E)** 2D ^1^H-^15^N sensitivity enhanced HSQC spectra of 0.4 mM ^15^N-labeled ChDode covalent dimer in water (pH 4.0, 30°C, 600 MHz, 2,048 × 128 data points, 16 × 24 ppm spectral width) and in DPC micelles (pH 4.0, 40°C, 800 MHz, 2,048 × 64 data points, 15 × 15 ppm spectral width, dimer-to-lipid molar ratio, *D*:*L* = 1:130). The obtained resonance assignments are shown. The resonances of side chain groups are shown in the inserts. Assignment of the resonances from the outer chains of the ChDode tetramer in DPC is shown in red and underlined. **(B,F)** The representative conformers of ChDode in water and DPC micelles in ribbon representation. The positively charged, aromatic, and polar residues are colored in blue, green, and magenta, respectively. Disulfide bonds and hydrophobic residues are colored in yellow. The chains of the dimer and tetramer are numbered. The residues from the second peptide chains of the ChDode dimers in water and DPC are denoted by asterisks. The residues from the outer chains of the ChDode tetramer in DPC is shown in red and underlined. **(C,D,G,H)** Electrostatic and molecular hydrophobicity (Pyrkov et al., 2009) potentials are shown on the molecular surfaces of the ChDode dimer in water **(C,D)** and the ChDode tetramer in DPC micelles (**G,H**, two-sided view). Red, blue, green, and yellow areas denote negative, positive, polar, and hydrophobic regions, respectively.

The presence of two cysteine residues in the ChDode structure implies two possible ways of the disulfide bonds formation leading to parallel or antiparallel dimerization (Figures 1C,D). Only one set of signals was observed in the ChDode NMR spectra (Figure 4A), which is compatible only with the antiparallel structure (Figure 1C), as in this case the two polypeptide chains forming the dimer have identical conformation and chemical environment. On the contrary, in parallel beta-structural covalent dimer (Figure 1D) the two polypeptide chains have different chemical environment and conformation and thus must exhibit different chemical shifts.

The temperature dependence of HN protons chemical shifts (Δδ^1^HN/ΔT) permits to characterize the pattern of backbone-backbone H-bonds in polypeptides. It is usually assumed that HN groups with |Δδ^1^HN/ΔT| <4.5ppb/K participate in the intramolecular H-bonds (Cierpicki and Otlewski, 2001). The data obtained for ChDode in water (Supplementary Figure 6A) showed that the HN groups in the even-numbered residues are probably involved in the H-bonds formation. Such H-bond pattern is indicative of the antiparallel β-structure. The formation of the continuous β-strand by the ChDode monomeric unit within the dimer is also confirmed by the large values of ^3^J_*H*_^*N*^_*H*_^*A*^ spin-spin couplings (Supplementary Figure 6A), and by non-interrupted chain of strong HN(i+1)-Hα(i) NOE cross-peaks (Supplementary Figure 6B). The antiparallel pairing of the β-strands in the ChDode homodimer was confirmed by the intermonomer NOE contacts: Arg1:Hγ-Cys11:Hβ3, Cys3:Hβ3-Arg9:Hγ3, Ile2:HN-Arg12:HN, Gln4:HN-Val10:HN, Phe5:Hα-Val10:HN, *etc.* (for example see Supplementary Figure 6B red).

The set of 20 spatial structures of the covalent ChDode homodimer was calculated from the obtained NMR data (Supplementary Figure 6C and Supplementary Table 2). The two-stranded antiparallel β-sheet in the homodimer is stabilized by two symmetric disulfide bridges (Cys3-Cys11) and by 12 intermonomer H-bonds (Figure 4B). The β-sheet in the ChDode dimer has a significant right-handed twist of 247° ± 5° (per 8 residues) and kink of 49° ± 2°. This distortion probably shields the hydrophobic cluster formed by Val6, Ile8, and Val10 side chains of the both monomers from contact with the polar solvent. These hydrophobic groups are located on the concave face in the central part of the ChDode dimer. The presence of the distortion in the ChDode β-structure is in agreement with the results obtained by CD spectroscopy (see above).

The analysis of molecular surface properties (Figures 4C,D) revealed, that all the charged and polar groups (Arg1, Gln4, Arg9, Arg12, *N*-, and *C*-terminal groups) are situated in the terminal regions of the dimer, while the central region is enriched with the hydrophobic and aromatic residues. In addition to the aforementioned residues that form a concave face of the dimer, the Phe5 and Leu7 side chains are located on the convex face in the central region of the dimer (Figure 4D).

### NMR Structure of PcDode in the Aqueous Solution

The fact that ChDode in aqueous solution has the structure of a strongly twisted antiparallel β-structural homodimer (24 residues), stabilized by two disulfide bonds, raises the question of how this structure is characteristic for others cathelicidins-1 (products of *CATHL1* genes). To answer this question, we studied 3D structure of PcDode – cathelicidin-1 from sperm whale. The sequence of PcDode corresponds to doubled dodecapeptide sequence.

**FIGURE 5:**
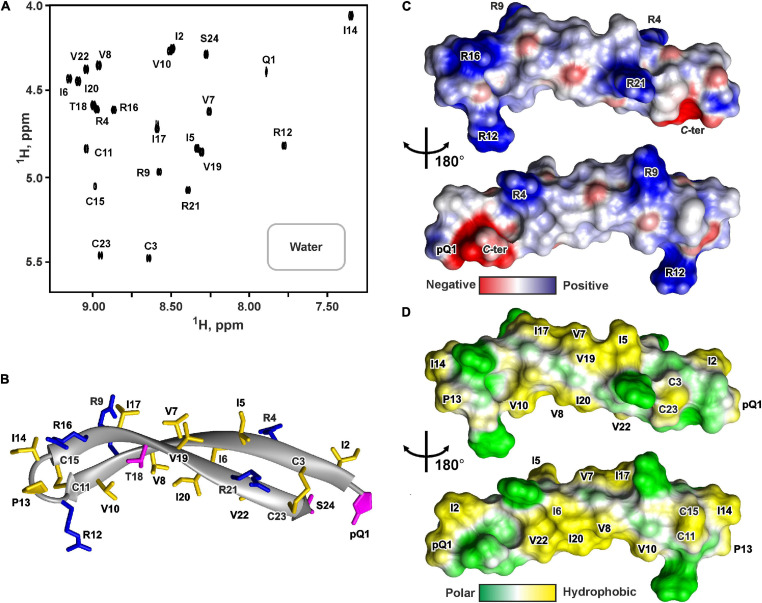
NMR data defines 3D structure of PcDode. **(A)** 2D ^1^H-TOCSY spectrum (mixing time – 80 ms) of 0.6 mM PcDode in water (pH 4.4, 30°C, 600 MHz, 8,192 × 600 data points, 18 ppm × 11 ppm spectral width). The obtained resonance assignments are shown. **(B)** The representative conformer of PcDode in ribbon representation. The positively charged and polar residues are colored in blue and magenta, respectively. Disulfide bonds and hydrophobic residues are colored in yellow. Electrostatic **(C)** and molecular hydrophobicity (Pyrkov et al., 2009) **(D)** potentials are shown on the molecular surfaces of the PcDode. Red, blue, green, and yellow areas denote negative, positive, polar, and hydrophobic regions, respectively.

Similarly to ChDode, the PcDode peptide also contains only one acidic group – the *C*-terminal carboxyl. Therefore, we used slightly acidic pH (4.4) for the NMR analysis. Under these conditions, the ionization state of peptides should be close to the state at neutral pH 7.0 (total charge of the peptide–+4). In contrast to ChDode, the N-terminus of PcDode molecule is probably deprived of the positive charge because Gln1 residue can interconvert into cyclic pyroglutamate. The presence of this posttranslational modification was confirmed by the obtained NMR data and especially by observation of HN resonance for the N-terminal residue (Figure 5A).

The set of 20 PcDode structures was calculated based on NMR data obtained for the unlabeled recombinant peptide variant (Supplementary Figure 7). The peptide represents β-hairpin, formed by two antiparallel β-strands (Ile2-Cys11 and Cys15-Cys23) (Figure 5B). The tip of the β-hairpin (Arg12-Ile14) forms a turn involving the *cis* Arg12-Pro13 peptide bond. The *cis* configuration of this bond was supported by the observed Arg12Hα-Pro13:Hα NOE contact. The PcDode structure is stabilized by two intramolecular disulfide bonds (Cys3-Cys23 and Cys11-Cys15) and ten backbone-backbone hydrogen bonds (Figure 1F). Similarly to ChDode, the two-stranded β-sheet of PcDode has a pronounced right-handed twist of 199° ± 24° (per 8 residues) and kink of 36° ± 2° (Figure 5B). The analysis of molecular surface properties (Figures 5C,D) revealed localization of the charged and polar groups (pGln1, Arg4, Arg9, Arg12, Arg16, Arg21, Ser24, and *C*-terminal group) in the terminal regions of the prolonged β-hairpin. At the same the central region is enriched with the hydrophobic residues. The Ile6, Val8, Ile10, Ile20, and Val22 side chains are located on a concave face of the kinked β-structure, while the Ile5, Val7, Ile17, and Val19 side chains form a hydrophobic patch on a convex face (Figure 5D).

### Secondary Structure of ChDode in Detergent Micelles and Lipid Vesicles: CD and FTIR Spectroscopy

Two types of small unilamellar vesicles (SUVs) were used to investigate changes in the ChDode structure upon interaction with lipid membranes. The first lipid system was based on the zwitterionic soybean PC and mimicked the plasma membrane of eukaryotic cell, while the second system (2:1 mixture of soybean PC and anionic DMPG) was used to model bacterial membranes. Micelles of zwitterionic detergent dodecyl-phosphocholine (DPC) were also used to investigate the secondary structure of ChDode in the anisotropic membrane-mimicking environment.

The CD spectra of ChDode in lipid vesicles and detergent micelles (Figure 3A) had appearances similar to the spectrum in ethanol solution. In all cases, two major peaks were observed: positive at 190–200 nm and negative at 210–220 nm. An additional weak positive peak, probably related to the contributions of Phe5 or disulfide bridge, was observed at 234 nm in lipid vesicles. Despite the fact that the position and intensity of the major CD peaks varied significantly, the obtained spectra revealed the preservation of the β-structural organization of ChDode upon interaction with lipid vesicles and DPC micelles.

Significant changes were observed in the peptide FTIR spectra upon transition from isotropic to anisotropic environments. In addition to three bands (at ∼1,625, ∼1,675, and ∼1,695 cm^–1^) observed in water and ethanol (Figure 3B), the amide I contour in PC/DMPG membranes and DPC micelles contained an additional major band at ∼1,650 cm^–1^ (Figure 3C). These data confirmed preservation of the antiparallel β-structure by the ChDode dimers but, at the same time, revealed formation of additional H-bonds with properties different from those of the intramolecular H-bonds in the covalent ChDode dimer. Most probably, the peptide dimers oligomerize in the charged PC/DMPG membrane and DPC micelles with formation of extended β-sheets stabilized by ‘new’ interdimer H-bonds. These spectra are similar to the FTIR spectrum of the β-hairpin AMP arenicin (Figure 3C, cyan dash dot doted line), which, according to our previous data, forms β-structural oligomers in the PC/PG membrane (Shenkarev et al., 2011). The FTIR spectrum of ChDode in zwitterionic PC vesicles (Figure 3C, magenta longdashed line) represents a combination of the spectrum of dimer in water (Figure 3B) and the spectrum of oligomers in PC/DMPG vesicles or DPC micelles. Thus, at the dimer-to-lipid molar ratio (D:L) of 1:120, the ChDode dimers in solution are in equilibrium with the oligomers bound to the PC membrane. The spectral decomposition showed that the molar ratio of the covalent dimers to oligomers in the sample was of 2:1.

The spectrum corresponding to the oligomers was also observed in the partially anionic DMPE/DMPG (2:1) vesicles at low ionic strength (10 mM NaPi plus 10 mM NaCl) (Figure 3D, red line). This lipid system was used for measurement of the ChDode proton transfer activity (see section “Hydrodynamic Properties and Topology of the ChDode/DPC Micelle Complex”). Both these peak positions and intensities were close to those observed in the PC/DMPG vesicles (Figure 3C). At the same time, an increase in the NaCl concentration to 1 M led to the appearance of the spectrum (Figure 3D, thick blue dashed line) that resembled the ChDode spectrum measured in PC membranes (Figure 3C) and was a superposition of the spectra of the dimer and oligomers. Thus, shielding of the electrostatic interactions at high ionic strengths resulted in a partial dissociation of the ChDode oligomers to the dimers.

### NMR Structure of the ChDode Tetramer in DPC Micelles

Two types of detergent micelles were used to investigate ChDode structure in the membrane-mimicking environment: the micelles of anionic lysolipid LMPG and zwitterionic micelles of DPC. Gradual addition of LMPG to the ChDode sample resulted in the peptide aggregation accompanied by significant broadening of the peptide NMR signals. An increase in the LMPG concentration up to the *D*:*L* ratio of 1:130 did not lead to the narrowing of the ChDode resonances; therefore, the NMR spectra remained unanalyzable. Thus, negatively charged LMPG molecules caused strong oligomerization of the ChDode dimers, which had a significant positive charge (+6).

At the same time, an addition of small amounts of DPC (below the critical-micelle-concentration) to the ChDode sample (*D*:*L* ∼ 1:3.5, [DPC] ∼ 1.4 mM) resulted in slight broadening and changes in the positions of the NMR signals from the central part of the ChDode dimer (Supplementary Figures 8A–C). The signals of the Val6, Ile8, and Val10 residues belonging to the concave hydrophobic face of the dimer were most sensitive to the DPC addition. Thus, monomeric DPC molecules initially interact with this cavity on the ChDode surface and do not disrupt the dimeric structure. A further increase in DPC concentration led to significant broadening of the peptide signals at *D*:*L* above 1:16, followed by an appearance of a specific spectral pattern characterized by a double number of ^1^H-^15^N cross-peaks at *D*:*L* above 1:60 (Figure 4E). This revealed the formation of the ChDode tetramers (non-covalent dimers of covalently linked homodimers) in complex with DPC micelle (Figure 1E). The tetramer of the peptide has *C*_2_ symmetry, but the ‘outer’ (#1 and #4) and ‘inner’ (#2 and #3) peptide chains have different environments and therefore their chemical shifts are different (Supplementary Figure 8D). A further increase in the detergent concentration resulted in the gradual narrowing of the peptide signals with ^1^H^*N*^ line-width going into a plateau at *D*:*L* ratios above 1:100. Finally, the dimer-to-lipid(detergent) molar ratio of 1:130 was chosen for structural study of the ChDode tetramer (Figure 4E). The signal broadening observed at low *D*:*L* ratios could be a consequence of μs-ms time scale exchange process between different structural states of the peptide (e.g., dimer in solution, dimer in complex with the micelle, tetramer in complex with the micelle, and higher order aggregates) which had an intermediate rate on the NMR time scale.

Almost complete backbone and side chain assignments were obtained for both sets of NMR signals. The dimerization of the covalent homodimers (formation of tetramers) was confirmed by observation of NOE contacts between ‘inner’ chains, which cannot be satisfied within the single β-strand (Phe5:HN-Arg9:HN, Phe5:HN-Val10:HA, and Gln4:Hε-Cys11:HN). As expected for β-structural tetramer containing four strands, no long-range NOE contacts were observed between ‘outer’ chains. The temperature gradients of amide protons (Supplementary Figure 6D) also confirmed the assignment of the two resonance sets to the ‘outer’ and ‘inner’ peptide chains within the tetramer. In both cases the HN groups in the even-numbered residues demonstrated a low amplitude of the temperature gradients indicating H-bonds formation. This confirms the preservation of the β-structural conformation by the covalent homodimers of ChDode upon association with DPC micelle and further dimerization. At the same time, the Δδ^1^HN/ΔT values revealed the participation of the HN groups of the Phe5, Leu7, and Arg9 residues from the ‘inner’ chains in the H-bond formation (Supplementary Figure 6D). Thus, the tetrameric assembly of ChDode in the DPC micelles environment is stabilized by six interdimer H-bonds, while each covalent dimer is stabilized by two disulfide bridges and by 12 intermonomer H-bonds (Figure 1E).

The set of 20 spatial structures of the ChDode tetramer was calculated from the obtained NMR data (Supplementary Figure 6E and Supplementary Table 2). As compared with the dimer structure in water (Supplementary Figure 6C), the four-stranded β-sheet of ChDode in DPC micelles adopts much flatter structure. The right-handed twist of the two identical dimers within the tetramer was reduced to 78° ± 16° (per 8 residues) and the angle of a kink was reduced to 24° ± 3° (Figure 4F). At the same time, the relative orientation of the two dimers in the resulting tetrameric structure is not well defined (Supplementary Figure 6F). In the obtained set of structures, the cross angle of two dimers is varied from −37° to +15° with an average of −29° ± 11°.

Flattering of the ChDode β-structure upon transfer into the micelle and dimerization resulted in formation of a brick-like (rectangular parallelepiped) structure, with the dimensions of about 45 Å × 25 Å × 15 Å (Figures 4F–H). The structure has amphipathic properties. Two regions around smaller faces of the parallelepiped accommodate all charged and polar groups from the four ChDode monomers (Arg1, Gln4, Arg9, Arg12, *N*-, and *C*-terminal groups). Two hydrophobic clusters are situated on the largest faces of the parallelepiped. The largest of them accommodates side chains of Ile2, Val6, Ile8, and Val10 from all four monomers, while the second cluster is formed on the opposite face by the side chains of Phe5 and Leu7. Together, these clusters form a hydrophobic belt that runs through the long edges of the parallelepiped (Figure 4H).

### ^15^N-Relaxation Data Revealed Decrease in Backbone Mobility of ChDode Upon Incorporation Into DPC Micelle and Dimerization

**FIGURE 6:**
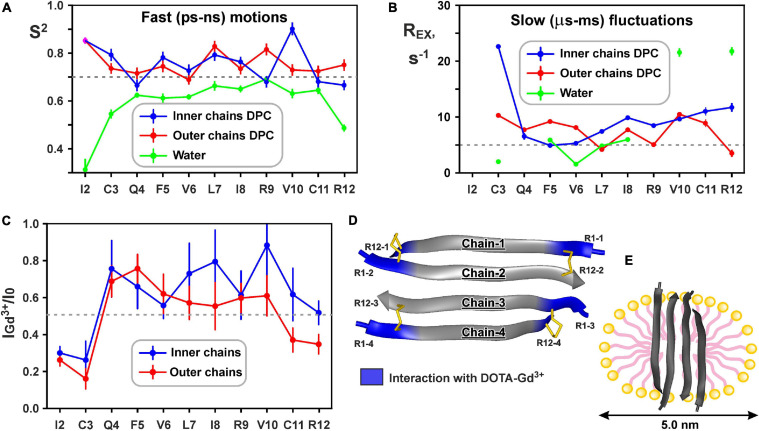
Changes in backbone dynamics of ChDode upon incorporation into DPC micelle and dimerization and topology of the ChDode/DPC micelle complex. **(A)** The values of generalized order parameters (*S*^2^) calculated during ‘model-free’ analysis of ^15^N relaxation data. The *S*^2^ values correspond to amplitude of motions at ps-ns timescale. The lower *S*^2^ value, the greater amplitude of motions. Residues displaying *S*^2^ < 0.7 are subjected to extensive motions in ps-ns timescale. In relaxation spectra the ^1^H-^15^N signals of Ile2 residues from inner and outer chains in DPC micelles were overlapped. **(B)** The values of exchange contributions to the *R*_2_ relaxation rates (*R*_*EX*_). The non-zero *R*_*EX*_ values (cut-off value of 5 s^– 1^ is shown) indicate motions at μs-ms timescale. **(C)** Attenuation of the HN cross-peaks in the ^15^N-HSQC spectrum of the ChDode tetramer by water-soluble paramagnetic DOTA-Gd^3+^ complexes. The 0.5 threshold line subdivides data points in two groups: the points below the line corresponds to residues with HN-groups accessible to solvent, the points above the line corresponds to HN-groups shielded from the paramagnetic probe in the micelle interior. **(D)** Data mapped on the structure of the ChDode tetramer. **(E)** Proposed model of the ChDode/DPC complex.

The large hydrophobic regions on the peptide surface are responsible for the favorable interaction of the ChDode covalent homodimer with membrane and membrane mimicking micelles. At the same time, the efficiency of the peptide-membrane interactions could also depend from the mobility of a peptide molecule. A decrease in the mobility of a peptide when it is incorporated into a membrane (micelle) can lead to a decrease in entropy and an unfavorable contribution (−*T*⋅Δ*S*) to the free energy of the membrane binding (Δ*G*). To estimate the magnitude of this effect we characterized the peptide backbone dynamics in water and DPC micelles by ^15^N-relaxation measurements. The ^15^N-relaxation data measured at 80 MHz and results of their analysis are presented in Supplementary Figure 9. The Figures 6A,B show parameters describing ‘fast’ ps-ns motions (generalized order parameters, *S*^2^) and ‘slow’ μs-ms conformational fluctuations (exchange contributions to the *R*_2_ relaxation rate, *R*_*EX*_), respectively.

Low values of the squared order parameters *S*^2^ (average of 0.59 ± 0.11) revealed a high backbone mobility in the covalent ChDode homodimer at the ps-ns time scale (Figure 6A, green). The characteristic time of these motions (τ_*E*_) was of 69 ± 23 ps (Supplementary Figure 9), while its amplitude (shown by *S*^2^ values) increased from the central region toward the *N*- and *C*-termini of the peptide (Figure 6A). At the same time, the micelle incorporation and dimerization significantly diminished the amplitude of motions at this timescale. An average *S*^2^ value was increased to 0.74 ± 0.08 and 0.75 ± 0.04 for ‘inner’ and ‘outer’ chains, respectively (the *S*^2^ value for overlapped Ile2 signal was not accounted) (Figure 6A, blue and red). Interestingly, the characteristic time of these motions was not changed significantly (τ_*E*_ = 23 ± 10 ps, Supplementary Figure 9). Using formulae from Yang and Kay (1996) we can convert observed changes in *S*^2^ values to entropy change (Δ*S*). Results indicate that stabilization of the ChDode backbone on the picosecond to nanosecond time scale upon incorporation into DPC micelles and dimerization can results in a large energy penalty (16 kcal/mol per 4 × 12 residues) to the free energy of membrane binding.

In contrast to distribution of ps-ns motions, the μs-ms mobility was observed in the central and C-terminal regions of the ChDode molecule in water (Figure 6B, green). The highest *R*_*EX*_ values (>20 s^–1^) was observed for HN groups of Val10 and Arg12 residues, whose side chains are at the concave face of the ChDode dimer. Considering that Arg12 is the only charged residue belonging to the hydrophobic concave face of the dimer, we suggest that the observed μs-ms motions are associated with the fluctuations in the relative packing of the Val10 and Arg12 side chains. The distribution of μs-ms motions changed significantly upon transfer to the DPC micelle and dimerization (Figure 6B, blue and red). All ChDode residues from outer and inner chains of the tetramer demonstrated non-zero *R*_*EX*_ values and the largest value (*R*_*EX*_ ∼ 23 s^–1^) was observed at the *N*-terminus (Cys3 residue) of the inner tetramer chain. Most likely, the μs-ms fluctuations observed in DPC micelles are associated with the dimer-tetramer equilibrium or with the equilibrium between the tetramer and higher-order aggregates. Despite the fact that the concentrations of ChDode dimers and higher-order aggregates are negligible under the experimental conditions used, their presence can still be detected via the relaxation measurements. It should be noted, however, that magnitude of *R*_*EX*_ contributions is not directly proportional to the amplitude of motions or number of accessible conformational states. In other words, the observed differences in distribution of *R*_*EX*_ values may not be associated with the significant entropy change.

### Hydrodynamic Properties and Topology of the ChDode/DPC Micelle Complex

^15^N relaxation data also provide information about hydrodynamic properties of proteins and peptides in solution. The correlation time of the overall rotational diffusion of the ChDode covalent homodimer in water (τ_*R*_ = 2.1 ns) calculated from experimental data was slightly lower than the corresponding value (2.7 ns) predicted by hydrodynamic calculations starting from the determined 3D structure. This confirms the absence of oligomerization of the ChDode dimers in aqueous solution. In contrast, rotational diffusion of the ChDode tetramer in complex with DPC micelle is characterized by correlation time τ_*R*_ of 9.4 ns. This value significantly exceeds the value predicted by hydrodynamic calculations for the ChDode tetramer (τ_*R*_ ∼4.0 ns). The experimental τ_*R*_ value corresponds to the hydrodynamic radius *R*_*H*_ ∼ 24.6 Å, which is slightly larger than the hydrodynamic radius of a pure DPC micelle (*R*_*H*_ ∼ 23 Å). This indicates that the ChDode tetramer is fully incorporated into the micelle and does not participate in additional overall diffusion-like motions within the peptide/detergent complex. Interestingly, in both cases the hydrodynamic calculations predict the anisotropy of the rotational diffusion tensor (axially symmetric, prolate, with Dz/Dx/Dy ratio about 2:1:1). Nevertheless, all HN vectors in the β-structure lie approximately in one (*xy*) plane defined by the principal axes of the diffusion tensor. Therefore, the diffusion anisotropy cannot be deduced from the ^15^N relaxation data.

Topology of the peptide/micelle complex was probed by an addition of paramagnetic DOTA-Gd^3+^ complexes (Figure 6C). The ChDode groups exposed to water had increased *R*_2_ relaxation rates due to contacts with the paramagnetic probe, and as a result their signals were attenuated. The observed attenuation of the signals of Ile2 and Cys3 residues of the outer and inner chains of the ChDode tetramer, and Cys11 and Arg12 residues of the outer chains showed that the tetramer termini are exposed to the solvent (Figures 6C,D). The central part of the ChDode tetramer is probably shielded from the paramagnetic probe in the micelle interior. Thus, the ChDode/DPC complex could be envisaged as an ellipsoidal or spherical particle (diameter ∼5 nm), in which the hydrophobic belt on the tetramer surface is coated with a layer of the detergent molecules (Figure 6E).

### Measurements of ChDode Proton Transfer Activity

**FIGURE 7:**
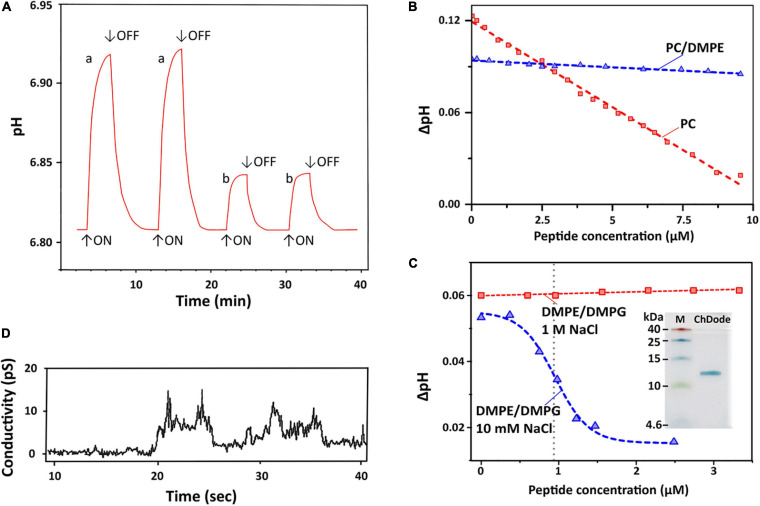
Proton transfer activity and permeabilization of planar lipid membrane. **(A)** Time course of light-induced pH changes in the unbuffered suspension (1 M NaCl, 25°C) of BR-proteoliposomes prepared from soybean PC without (a) and after an addition of 7 μM ChDode dimer (b). The arrows indicate time when illumination is turned on or off. Minimum 3 min pause after the peptide addition is necessary to reach the pH equilibrium and obtain reproducible results. **(B)** Dependence of light-induced pH changes (ΔpH) on the dimeric peptide concentration in PC (red) and PC/DMPE (1:1, blue) liposomes (1 M NaCl). **(C)** Dependence of light-induced ΔpH values on the dimeric peptide concentration in DMPE/DMPG (2:1) liposomes at two NaCl concentrations (10 mM and 1 M). The Hill equation (*y* = B0 + (A0 – B0)/(1 + ([ChDode]/IC_50_)^*nH*^)) was fitted to the data obtained at 10 mM. The calculated IC_50_ and nH parameters were 0.95 ± 0.03 μM and 4.6 ± 0.6. Insert: Tricine-SDS-PAGE of the recombinant ChDode (0.5 μg). M, molecular mass marker. **(D)** Currents through a lipid bilayer membrane made of polar lipid extract from *E. coli* induced by 1.67 μM ChDode dimer (5 mM HEPES, 20 mM NaCl, pH 7.4, applied voltage 50 mV). A heterogeneous population of conductance was observed.

To characterize a possible mechanism of ChDode membranotropic action, lipid-dependent pore formation was monitored by measurements of the proton transfer activity (PTA). Protonophore activity was analyzed using proteoliposomes containing proton pump – bacteriorhodopsin from *H. halobium* (BR) (Bayley et al., 1982). After incorporation into lipid vesicles, BR exhibits inward light-induced proton translocation which causes an increase in the pH of the external medium (Sychev et al., 2015). An addition of the peptide influenced the membrane barrier function and reduced the proton concentration difference, lowering the pH value of the bulk solution to a certain level. Figure 7A represents typical traces for light-induced pH changes.

Dependence of bulk pH values on a concentration of added ChDode for three membrane systems is presented on the Figures 7B,C. Linear dependence of ΔpH on a total concentration of the peptide in zwitterionic PC membranes indicates that ChDode does not form oligomeric pores in this bilayer. As mentioned above, the ChDode dimers in solution are in equilibrium with the oligomers bound to the PC membrane at high (in the mM range) concentration used for FTIR measurements (Figure 3C). Evidently, at lower concentrations (in the μM range) used for PTA measurements the oligomerization might be significantly reduced. Thus, the proton transfer observed in the PC membranes was probably caused by the individual ChDode covalent dimers. A very gentle slope of the ΔpH concentration dependence in PC/DMPE (1:1) membranes (Figure 7B) shows that ChDode has a very weak activity in liposomes composed of these two zwitterionic lipids. Most probably, a negative curvature strain, induced by DMPE, prevents deep incorporation of the ChDode dimers into the membrane, or prevents the peptide transition from the surface-bound to the transmembrane state.

For liposomes composed of DMPE/DMPG (2:1, 10 mM NaCl) (Figure 7C), the measured ΔpH[c] dependence can be described by the sigmoidal function characterized by IC_50_ ∼ 0.95 μM and Hill’s coefficient (nH) ∼ 4.6. This indicates that the observed proton transfer activity is caused by oligomeric pores composed from four to five covalent dimers of ChDode. The Tricine-SDS-polyacrylamide gel electrophoresis (Figure 7C insert and Supplementary Figure 4) also showed that the peptide in the presence of anionic lipid (SDS) is prone to oligomerization and is capable of forming multimers, including those consisting of eight β-strands or four covalent dimers (12 kDa).

Interestingly, the proton transfer was not observed at high ionic strength (1 M NaCl), i.e., the peptide does not work when electrostatic interaction with the negatively charged component (DMPG) of the membrane is impaired. As shown above by FTIR (Figure 3D), the increase in NaCl concentration led to dissociation of ChDode oligomers and appearance of the covalent dimer fraction. It is likely that the blockade of electrostatic interactions with 1 M NaCl made the properties of DMPE/DMPG bilayer very close to those of the PC/DMPE system. The absence of PTA in the DMPE/DMPG and PC/DMPE systems (both at 1 M NaCl) allowed to use these data as a control, showing that ChDode by itself did not inhibit the BR activity.

### ChDode Induces Conductance in Planar Lipid Membranes

Figure 7D shows currents through a planar lipid bilayer mimicking the plasma membrane of Gram-negative bacteria induced by ChDode at low NaCl concentration (20 μM). This lipid system consists of PE, PG, and DPG and, therefore, resembles DMPE/DMPG (2:1) membranes used for PTA measurements (see above). The observed conductance revealed the formation of heterogeneous population of ion-permeable pores having a relatively low conductivity in the range from 10 to 60 pS. Probably, the toroidal pores with the different conductance were formed by various multimers of ChDode in complex with anionic lipids (PG or DPG). The lifetimes of the open and closed states were in the seconds time range. The BLM membrane was not disrupted by the ChDode addition.

The general conclusion from study of the peptide-induced conductance in model membranes is that ChDode can form proton pores in liposomes and narrow pores in planar BLM without membrane disruption. The same activity, including formation of stable pores, was shown for the β-hairpin peptide arenicin in our previous work (Shenkarev et al., 2011; Sychev et al., 2017b).

### Cathelicidin-1s Biological Activity and Mechanism of Action

Amphiphilic AMPs are known to be adsorbed on plastic surfaces (Wiegand et al., 2008). To minimize this effect, serial dilutions of the peptides were performed in the presence of 0.05% BSA in the growth medium. Antibacterial activity of ChDode was determined using a two-fold serial dilution assay in MH medium with or without addition of NaCl to the physiology concentration.

The minimum inhibitory concentrations (MICs) of cathelicidins against Gram-positive and Gram-negative bacteria are presented in Table 1. ChDode and PcDode showed a moderate antimicrobial effect against bacterial strains tested. As compared with other cathelicidins, the effects were more pronounced against Gram-negative bacteria. However, the presence of 0.154 M NaCl resulted in several-fold decrease their activity against all the strains tested. A similar negative effect of salt on antimicrobial activity has been shown for several other cathelicidins. It has been shown that salt might inhibit an absorption of the peptides on the bacterial surface (Gennaro et al., 2002).

**TABLE 1:** Antibacterial activity of goat cathelicidins and whale cathelicidin-1 PcDode.

Strain	Minimum inhibitory concentration (μM)*							
	ChDode		PcDode		ChMAP-28		Mini-ChBac7.5Nα	
	Without NaCl	0.154 M NaCl	Without NaCl	0.154 M NaCl	Without NaCl	0.154 M NaCl	Without NaCl	0.154 M NaCl
***E. coli* ML-35p**	8	16	16	32	0.06	0.06	0.5	4
***S. aureus* 209P**	16	>64	32	>64	0.06	0.5	2	16

*Antibacterial testing was performed in the Mueller-Hinton broth at 37°C.

**FIGURE 8:**
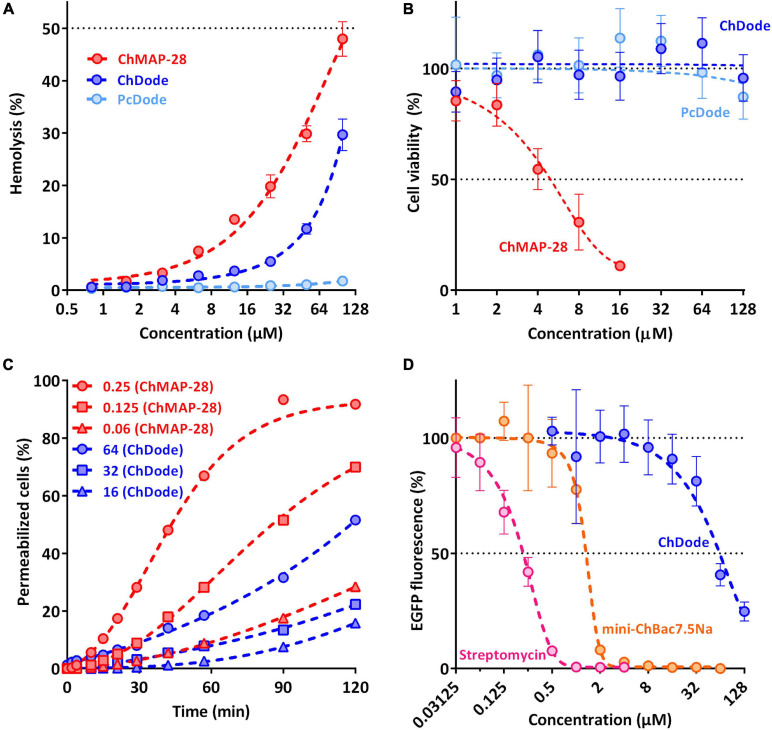
Biological activity of cathelicidins. **(A)** Hemolytic activity of the peptides after 1.5 h incubation (hemoglobin release assay). **(B)** Cytotoxicity of the peptides toward human keratinocytes (HaCaT) cells after 24 h incubation (MTT-assay). **(C)** Kinetics of changes in *E. coli* ML-35p cytoplasmic membrane permeability (ONPG assay). **(D)** Effects of goat cathelicidins and streptomycin at different concentrations on the fluorescence resulting from *in vitro* translation of EGFP with the use of *E. coli* BL21 (DE3) Star cell extract. Data are the mean ± SD of at least three independent experiments performed in triplicate.

The main mechanism of action on bacterial cells for most β-hairpin AMPs is disruption of membrane integrity. An influence of the goat cathelicidins on *E. coli* ML-35p membrane was characterized by monitoring the membrane permeability to chromogenic marker – *o*-nitrophenyl-β-D-galactoside (ONPG). The membranolytic peptide melittin was used as a positive control. ChDode showed a weak activity against membranes, which did not grow much with increasing of the peptide concentration (see Figure 8C). At the MIC value (16 μM), ChDode caused membranes damage in less than 20% of the cells. At the concentration of 64 μM (4 × MIC) the caused membranes permeabilization in more than 50% of the cells. In comparison, the membrane-active peptide ChMAP-28 at concentrations of 0.06 and 0.125 μM caused permeabilization of 30 and 75% of the cells, respectively. Thus, ChDode acts on membranes but does not lead to their destruction.

To estimate cytotoxic effects of peptides, human red blood cells (hRBC) as well as adhesive cell lines of human keratinocytes (HaCaT) were used. ChMAP-28 which is active against any biological membranes, was used as a positive control. The mini-ChBac7.5Nα peptide has very low toxicity, as previously shown (Panteleev et al., 2018). The effect of ChDode on the erythrocyte membrane is much less pronounced than that of ChMAP-28 (see Figure 8A). The ChDode concentration causing lysis of 10% of red blood cells was of about 50 μM. For the ChMAP-28 peptide, this value was of 10 μM. These concentrations exceed the MIC values against *E. coli* by 3 and >100 times for ChDode and ChMAP-28, respectively. Therefore, ChDode has a relatively small therapeutic index, which indicates a low specificity of its membranotropic effect. The PcDode demonstrated an almost complete absence of hemolytic activity. Both cathelicidins-1 were shown to be non-toxic against human keratinocytes, even at the maximum concentration of 128 μM, while ChMAP-28 has IC_50_ against HaCaT cells of 5.4 ± 1.38 μM (see Figure 8B).

Next, we also tested an ability of ChDode to inhibit protein biosynthesis *in vitro* (see Figure 8D). The experiment was carried out using the bacterial cell-free protein synthesis system expressing the enhanced green fluorescent protein (EGFP). The results obtained with the use of streptomycin, displaying IC_50_ value of 0.2 μM and a full inhibition at the concentration of >1 μM corresponded with the published data (Krizsan et al., 2014). The obtained IC_50_ value for mini-ChBac7.5Nα was also consistent with previous data (Panteleev et al., 2018). ChDode showed a low ability to inhibit protein biosynthesis. The concentrations at which inhibition occurs significantly exceed its MIC values and may result from peptide aggregation at high concentration range. The IC_50_ value was of 54.95 ± 5.04 μM, while the MIC for the same strain was of 16 μM. Apparently, inhibition of protein biosynthesis might be caused by non-specific binding of the peptide to nucleic acids, as has been shown for some other AMPs with a similar structure. A similar level of inhibition was shown for tachyplesin-1 (Panteleev et al., 2018), which is known to be bound to DNA (Yonezawa et al., 1992). Thus, we can assume that ChDode mainly acts on the cytoplasmic membrane by increasing its permeability.

### Study of Synergy Between Different Goat Cathelicidins

**FIGURE 9:**
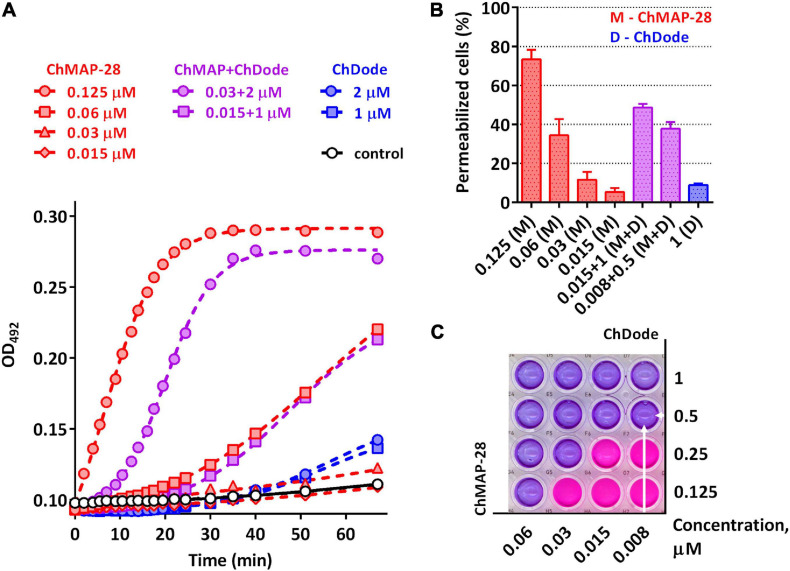
**(A)** Kinetics of changes in *E. coli* ML-35p outer membrane permeability measured with the use of chromogenic marker – the products of nitrocefin (OD_492_) hydrolysis. Comparative analysis of outer membrane permeability resulting from incubation with individual ChMAP-28, ChDode at various concentrations and their combination. Three independent experiments were performed, and the curve pattern was similar for the three series. **(B)** The ONPG testing for permeability of the cytoplasmic membrane of *E. coli* ML-35p. The graph shows effects of the peptides at different concentrations after 2 h. Data are the mean ± SD of two independent experiments. **(C)** The effect of the combined action of the peptides ChMAP-28 (M) and ChDode (D) against bacterial cells after incubation with resazurin. The arrows indicate the well with the lowest reproducible FICI. The result was reproduced in three independent experiments.

Being widely represented among the Artiodactyla order, catelicidin-1-like peptides, however, showed a low overall level of antibacterial activity. Therefore, it was decided to analyze biological activity of the ChDode peptide in combination with other known goat cathelicidins. As we have shown earlier, cathelicidins are able to enhance each other’s action when being used together. Such a synergistic effect was demonstrated for the ChMAP-28 and mini-ChBac7.5Nα peptides (Panteleev et al., 2018). In this study, the aforementioned peptides were selected for testing in combination with ChDode against Gram-positive (*S. aureus* 209P) and Gram-negative (*E. coli* ML-35p) bacteria in the medium containing a physiological concentration of NaCl (0.154 M). Interestingly, a pronounced synergistic effect was achieved for ChDode and ChMAP-28 mixed at different molar ratios (Figure 9A). The minimum fractional inhibitory concentration index (FICI) values are presented in the Table 2.

**TABLE 2:** Synergy between goat cathelicidins ChDode and ChMAP-28.

Strain	ChDode, μM			ChMAP-28, μM			Minimal FICI*	Synergy
	MIC_*A*_	[A]	FIC_*A*_	MIC_*B*_	[B]	FIC_*B*_		
***E. coli* ML-35p**	16	0.5	0.031	0.06	0.008	0.125	0.156	Yes
***S. aureus* 209P**	>64	8	<0.125	0.5	0.03	0.063	<0.188	Yes

*The estimation of synergistic effects between cathelicidins was performed by calculating the fractional inhibitory concentration index (FICI) according to the equation: FICI = FIC_*A*_ + FIC_*B*_ = [A]/MIC_*A*_ + [B]/MIC_*B*_, where MIC_*A*_ and MIC_*B*_ are MICs of individual peptides, while [A] and [B] are MICs of A and B when used together. A synergistic effect was defined at a FICI ≤ 0.5. The table shows the lowest value of FICI, reproduced in three independent experiments.

It was demonstrated that in the presence of far sub-inhibitory concentrations of ChDode the α-helical membrane-active ChMAP-28 exhibited an 8- to 16-fold increase in its activity. Thus, the value of the fractional inhibitory concentration for ChMAP-28 against *E. coli* drop to nanomolar range (8–16 nM). The pair PcDode and ChMAP-28 exhibited similar, albeit less pronounced, synergistic effects (0.25 < FICI < 0.5). Therefore, despite the lower activity, the PcDode has a mechanism of action similar to ChDode.

Since both ChDode and ChMAP-28 are membrane-targeting peptides, the data obtained may indicate that they boost this activity when acting together. To test this hypothesis, a comparative analysis of an ability of the cathelicidins and their combinations to disrupt the integrity of *E. coli* outer and cytoplasmic membrane was conducted in a wide range of concentrations. Notably, the peptide mixture showed a significant increase in the observed effects, greatly exceeding those of the individual peptides (Figure 9). In particular, the combination of the peptides corresponding the lowest FICI value against *E. coli* ML-35p (0.008 μM ChMAP-28 + 0.5 μM ChDode) caused damage of cytoplasmic membranes of ∼40% cells, while effects of the individual peptides were <10% at the minimum tested concentrations (0.015 μM ChMAP-28 or 1 μM ChDode).

The data presented in Figure 9A suggest ChMAP-28 damages the outer membrane much faster and more intensively in the presence of sub-inhibitory concentrations of ChDode than each peptide alone. Earlier, we have shown that ChMAP-28 at sub-inhibitory concentrations can promote translocation of ribosome-targeting mini-ChBac7.5Nα into the periplasmic space. The FIC values for the synergistic pair ChMAP-28 and mini-ChBac7.5Nα are 0.008 and 1 μM, respectively. Notably, ChDode at a concentration of 1–4 μM causes a greater increase in the permeability of the outer membrane than ChMAP-28 at concentrations up to 0.03 μM. At the same time, in contrast to ChMAP-28 it does not provide synergy in combination with mini-ChBac7.5Nα (FICI = 1) suggesting the different mechanism of membrane damage. The simultaneous use of three goat cathelicidins in a test against *E. coli* ML-35p allows to lower the fractional inhibitory concentration of ChMAP-28 to 4 nM with the FIC values of 1 μM for both ChDode and mini-ChBac7.5Nα.

## Discussion

Recent studies demonstrated that oligomerization of AMPs is a very important parameter in regards to their antimicrobial activity, selectivity and cytotoxicity. An assembled-state of molecules influences on solubility of AMPs and their resistance to proteolytic degradation. It is also expected that oligomerization of AMP in the membrane is required for efficient membrane disruption. That is why a propensity for dimerization is an important property of AMPs which has to be studied in great detail. However, AMP structures determined by high-resolution NMR spectroscopy in detergent micelles are often monomeric. This is due to the denaturing properties of detergents, which, unlike to membrane lipids, tend to disrupt the peptide-peptide interactions. Only a few oligomeric structures of helical AMPs were reported in detergent micelles, in particular, the potent analog of amphibian magainin MSI-78 forms antiparallel helical dimers in DPC micelles (Porcelli et al., 2006), and the chicken cathelicidin VK22 adopts the tetrameric helical structure which was also determined in the zwitterionic DPC environment (Saravanan and Bhattacharjya, 2011). There are also several examples of β-hairpin peptides which dimerize in micellar environment. For porcine protegrins the antiparallel dimers were reported (Usachev et al., 2015), while parallel dimerization was observed for arenicins isolated from marine polychaeta (Shenkarev et al., 2011). Interestingly, these structures were also determined in the DPC micelles. In terms of overall topology of the β-structure, the antiparallel arrangement of the ChDode tetramer identified in this work (Figures 1E, 4F) is more similar to structures of protegrins than to those of arenicins.

To obtain recombinant ChDode we selected a heterologous expression system with the use of thioredoxin A as a carrier protein. At first, thioredoxin has a size close to that of the cathelin-like domain in the ChDode precursor protein. In addition, the AMP sequence is located in the *C*-terminal part of the fusion protein that is common for natural cathelicidins. As the result, a homogeneous target peptide was obtained as a covalent dimer. Interestingly, the recombinant fusion protein was mainly accumulated in a monomeric form when expressed in the reducing environment of *E. coli* cytoplasm (Supplementary Figure 2). Apparently, the oxidation of intermolecular disulfide bonds occurred during the isolation and purification stages. The reduction of disulfide bonds led to the appearance of a band corresponding to the 12-residue monomeric form of ChDode with the molecular mass of 1.5 kDa (Supplementary Figure 4). Previous studies of the bovine bactenecin biosynthesis gave evidence that this peptide also can be produced *in vivo* by association of two monomers with the formation of the dimer, stabilized by two intermolecular disulfide bonds, rather than a monomeric β-hairpin (Storici et al., 1996). Covalent self-dimerization with the formation of an antiparallel homodimer is a reported stage of maturation for some AMPs, for example, θ-defensin (Selsted, 2004) and rattusin (Ji et al., 2018). Latter peptide forms a homodimeric scaffold in which the polypeptide chain folds as an antiparallel structure bonded by five intermolecular disulfide bridges. In present study, we demonstrated a further oligomerization of ChDode resulting in the appearance of a band with the molecular mass of 12 kDa that was corresponded to the association of four dimers or eight 12-residue strands (Figure 8D).

In addition, we found the gene encoding a unique cathelicidin-1 from the sperm whale *P. catodon* which contains four cysteine residues as well as a proposed Arg-Pro-Ile turn motif and, therefore, consists of two dodecapeptide building blocks (Figure 1F). This may constitute an indirect confirmation of the hypothesis that natural dodecapeptide cathelicidins have antiparallel orientation of 12-residue strands. An antiparallel orientation of β-strands in the ChDode structures was confirmed by NMR spectroscopy. In water, ChDode predominantly forms an antiparallel covalent dimer. However, as evidenced by FTIR and NMR spectroscopy, in membrane-mimicking environment a significant oligomerization of the peptide with the formation of large flat β-sheet structures occurred. Such a behavior of ChDode is similar to the β-hairpin AMP arenicin, which forms β-structural non-covalent dimers and higher order aggregates in the membrane (Shenkarev et al., 2011). Apparently, oligomerization is important for the biological activity of ChDode. It has been shown that the peptide forms oligomeric pores in DMPE/DMPG liposomes and planar membranes which mimic the plasma membrane of Gram-negative bacteria (Figures 7C,D). Notably, increase of NaCl concentration to 1 M resulted in the ChDode inactivation, i.e., the peptide lost its membrane activity when electrostatic interaction with the negatively charged component (DMPG) of the membrane was impaired. Thus, the three factors are necessary for the ChDode membrane activity: (1) the presence of negatively charged lipids (PG or DPG) in the membrane, (2) a low salt concentration, and (3) the presence of PE – the lipid with a negative spontaneous curvature.

The strong dependence of the ChDode membrane activity from electrostatic interactions with lipid membrane looks quite surprising. Indeed, the peptide contains high proportion of hydrophobic and aromatic amino acids (ca. 60%, eight from 12 residues), nevertheless, it weakly interacts with zwitterionic POPC vesicles (Figure 3C) and fully incorporates into the DPC micelles only at the dimer to lipid molar ratios above 1:100. Obtained data about ‘fast’ ps-ns dynamics (Figure 6A) provided clue to this riddle. The β-sheet of the ChDode dimer possesses high intramolecular mobility in water and binding to the membrane (mimicked by the DPC micelle) freezes the intramolecular motions. The rough estimates, taking into account only backbone motions, revealed relatively high entropic penalty (16 kcal/mol per ChDode tetramer) to the free energy of membrane binding associated with this freezing. This number can be compared with the favorable energy of formation of two backbone-backbone hydrogen bonds or transfer of four bulky Trp side-chains from water to membrane phase (McDonald and Fleming, 2016). Thus, increased mobility of ChDode covalent homodimer in water solution can explain its relatively weak membrane activity toward zwitterionic systems. The similar properties, high mobility in water solution and weak activity in the zwitterionic membranes, were previously described for β-hairpin AMP arenicin (Shenkarev et al., 2011), also containing ∼60% of hydrophobic residues.

In general, the pore-forming behavior of ChDode (Figure 7D) is similar to corresponding records for the bovine bactenecin in planar bilayer membranes composed of DPhPC and DPhPG (4:1) (Wu et al., 1999). For both peptides, conductance events with a variable magnitude were observed. Note that ChDode pores do not require a high voltage across the membrane (50 mV) to initiate a conductance. This agrees well with the transmembrane (fully inserted) topology of ChDode tetramer observed in the DPC micelles in the absence of external electric field. Taking into account strong dependence of the ChDode oligomerization and pore forming activity from the composition of lipid bilayer and absence of well-defined conductance levels (Figure 7D) we can describe the corresponding defects in the membrane as a toroidal or mixed peptide-lipid pores (Allende et al., 2005). In interpreting the results of PTA and formation of low-conductivity channels, we hold to the previously discussed view (Sychev et al., 2017a,b) that β-structural AMPs can act via a non-lytic mechanism. Indeed, in all our experiments liposomes were absolutely stable for many days after bactenecin collapsed the membrane potential. Thus, ChDode acts via this unique mechanism: it forms toroidal pores in the membrane, but does not induce micellization of the bilayer.

For both ChDode and PcDode, a modest antimicrobial activity was shown. It is also significantly reduced by a physiological concentration of NaCl. As goat leukocytes were shown to simultaneously express mRNA for a set of cathelicidins including ChDode (Zhang et al., 2014), we supposed that the peptide act synergistically with cathelicidin-3 (ChBac7.5) targeting the bacterial ribosome and/or membrane active cathelicidin-6 (ChMAP-28). Earlier, we have shown the synergistic effects when using ChMAP-28 and truncated form of ChBac7.5 in combination. Therefore, it was decided to study the combined effect of ChDode with these AMPs on bacterial cells.

Here, we report a strong synergy between ChMAP-28 and ChDode (see Figure 9). The combination of these two peptides appears to have an increased membrane-permeabilizing activity possibly due to the formation of the membrane-active peptide complex. A similar effect previously observed for some other combinations of membranotropic antimicrobial peptides, in particular, of human cathelicidin LL-37 and defensin (Nagaoka et al., 2000). It was also shown that the frog peptides PGLa and magainin-2 are able to form the peptide membrane-attacking complex (Zerweck et al., 2017).

Surprisingly, ChDode demonstrated increased permeabilization of the outer membrane, but did not show a synergistic effect with mini-ChBac7.5Nα. Therefore, unlike ChMAP-28 its main mechanism of action does not seem to involve the destruction of the membrane (see above). Apparently, the pores formed by ChDode in the membranes are too small enough for big molecules like proline-rich AMPs to penetrate through them.

Notably, the presence of ChDode boosts the kinetics of *E. coli* outer membrane permeabilization by ChMAP-28 and thus can additionally prevent antimicrobial resistance. In addition, this reduces the fractional inhibitory concentrations of ChMAP-28 as the most toxic component. These data indicate that the ChDode peptide could be rather like an adjuvant, enhancing an overall antibacterial effect of the AMP cocktail produced by goat leukocytes. Accordingly, ChDode may selectively potentiate the activity of ChMAP-28 which, in turn, restores the activity of ChBac7.5 targeting bacterial ribosome. PcDode also showed a synergistic effect with ChMAP-28, suggesting a similar mechanism of action for all cathelicidin-1-like peptides.

## Data Availability Statement

The datasets generated for this study can be found in online repositories. The names of the repository/repositories and accession number(s) can be found below: https://www.rcsb.org, 7ACE, https://www.rcsb.org, 7ACB, and https://www.rcsb.org, 7OSC.

## Author Contributions

IB, PP, ZS, and TO contributed to the conceptualization of the work. IB, PP, SeS, ZS, and TO designed the experiments, analyzed data, and wrote the manuscript. IB, PP, SeS, StS, PM, and MM performed the experiments. TO reviewed and edited the manuscript and supervised the whole project. All authors read and approved the final manuscript.

## Conflict of Interest

The authors declare that the research was conducted in the absence of any commercial or financial relationships that could be construed as a potential conflict of interest.

## Publisher’s Note

All claims expressed in this article are solely those of the authors and do not necessarily represent those of their affiliated organizations, or those of the publisher, the editors and the reviewers. Any product that may be evaluated in this article, or claim that may be made by its manufacturer, is not guaranteed or endorsed by the publisher.

## Footnotes

1
http://hollywood.mit.edu/GENSCAN.html

2
http://www.camp.bicnirrh.res.in/index.php

3
www.rcsb.org

## References

[B1] Allende D. Simon S. A. McIntosh T. J. (2005). Melittin-induced bilayer leakage depends on lipid material properties: evidence for toroidal pores. *Biophys. J.* 88 1828–1837. 10.1529/biophysj.104.049817 15596510 PMC1305237

[B2] Andrä J. Hammer M. U. Grötzinger J. Jakovkin I. Lindner B. Vollmer E. (2009). Significance of the cyclic structure and of arginine residues for the antibacterial activity of arenicin-1 and its interaction with phospholipid and lipopolysaccharide model membranes. *Biol. Chem.* 390 337–349. 10.1515/BC.2009.039 19199831

[B3] Bayley H. Höjeberg B. Huang K.-S. Khorana H. G. Liao M.-J. Lind C. (1982). “[10] Delipidation, renaturation, and reconstitution of bacteriorhodopsin,” in *Methods in Enzymology* Biomembranes Part I: Visual Pigments and Purple Membranes II, ed. L. Packer (Cambridge, MA: Academic Press), 74–81.

[B4] Beck R. M. D. Bininda-Emonds O. R. P. Cardillo M. Liu F.-G. R. Purvis A. (2006). A higher-level MRP supertree of placental mammals. *BMC Evol. Biol.* 6:93. 10.1186/1471-2148-6-93 17101039 PMC1654192

[B5] Berditsch M. Jäger T. Strempel N. Schwartz T. Overhage J. Ulrich A. S. (2015). Synergistic effect of membrane-active peptides polymyxin B and gramicidin S on multidrug-resistant strains and biofilms of *Pseudomonas aeruginosa*. *Antimicrob. Agents Chemother.* 59 5288–5296. 10.1128/AAC.00682-15 26077259 PMC4538509

[B6] Chakrabartty A. Kortemme T. Padmanabhan S. Baldwin R. L. (1993). Aromatic side-chain contribution to far-ultraviolet circular dichroism of helical peptides and its effect on measurement of helix propensities. *Biochemistry* 32 5560–5565. 10.1021/bi00072a010 8504077

[B7] Cierpicki T. Otlewski J. (2001). Amide proton temperature coefficients as hydrogen bond indicators in proteins. *J. Biomol. NMR* 21 249–261. 10.1023/A:1012911329730 11775741

[B8] Cole R. (2003). FAST-Modelfree: a program for rapid automated analysis of solution NMR spin-relaxation data. *J. Biomol. NMR* 26 203–213. 10.1023/A:1023808801134 12766418

[B9] Czaplewski L. Bax R. Clokie M. Dawson M. Fairhead H. Fischetti V. A. (2016). Alternatives to antibiotics-a pipeline portfolio review. *Lancet Infect. Dis.* 16 239–251. 10.1016/S1473-3099(15)00466-1 26795692

[B10] Fjell C. D. Hiss J. A. Hancock R. E. W. Schneider G. (2011). Designing antimicrobial peptides: form follows function. *Nat. Rev. Drug Discov.* 11 37–51. 10.1038/nrd3591 22173434

[B11] García de la Torre J. Huertas M. L. Carrasco B. (2000). HYDRONMR: prediction of NMR relaxation of globular proteins from atomic-level structures and hydrodynamic calculations. *J. Magn. Reson.* 147 138–146. 10.1006/jmre.2000.2170 11042057

[B12] Gennaro R. Zanetti M. Benincasa M. Podda E. Miani M. (2002). Pro-rich antimicrobial peptides from animals: structure, biological functions and mechanism of action. *Curr. Pharm. Des.* 8 763–778. 10.2174/1381612023395394 11945170

[B13] Greenfield N. Fasman G. D. (1969). Computed circular dichroism spectra for the evaluation of protein conformation. *Biochemistry* 8 4108–4116. 10.1021/bi00838a031 5346390

[B14] Grimsley G. R. Scholtz J. M. Pace C. N. (2009). A summary of the measured pK values of the ionizable groups in folded proteins. *Protein Sci.* 18 247–251. 10.1002/pro.19 19177368 PMC2708032

[B15] Hollósi M. Majer Z. Rónai A. Z. Magyar A. Medzihradszky K. Holly S. (1994). CD and Fourier transform ir spectroscopic studies of peptides. II. Detection of beta-turns in linear peptides. *Biopolymers* 34 177–185. 10.1002/bip.360340204 8142587

[B16] Jackson M. Mantsch H. H. (1995). The use and misuse of FTIR spectroscopy in the determination of protein structure. *Crit. Rev. Biochem. Mol. Biol.* 30 95–120. 10.3109/10409239509085140 7656562

[B17] Ji S. Yun H. Park G. Min H. J. Lee C. W. (2018). Expression and characterization of recombinant rattusin, an α-defensin-related peptide with a homodimeric scaffold formed by intermolecular disulfide exchanges. *Protein Express. Purification* 147 17–21. 10.1016/j.pep.2018.02.006 29454031

[B18] Kościuczuk E. M. Lisowski P. Jarczak J. Strzałkowska N. Jóźwik A. Horbańczuk J. (2012). Cathelicidins: family of antimicrobial peptides. A review. *Mol. Biol. Rep.* 39 10957–10970. 10.1007/s11033-012-1997-x 23065264 PMC3487008

[B19] Krizsan A. Volke D. Weinert S. Sträter N. Knappe D. Hoffmann R. (2014). Insect-derived proline-rich antimicrobial peptides kill bacteria by inhibiting bacterial protein translation at the 70S ribosome. *Angew. Chem. Int. Ed. Engl.* 53 12236–12239. 10.1002/anie.201407145 25220491

[B20] Kuzmin D. V. Emelianova A. A. Kalashnikova M. B. Panteleev P. V. Balandin S. V. Serebrovskaya E. O. (2018). Comparative in vitro study on cytotoxicity of recombinant β-hairpin peptides. *Chem. Biol. Drug Des*, 91 294–303. 10.1111/cbdd.13081 28815904

[B21] Lazzaro B. P. Zasloff M. Rolff J. (2020). Antimicrobial peptides: application informed by evolution. *Science* 368:eaau5480. 10.1126/science.aau5480 32355003 PMC8097767

[B22] Lee J.-U. Kang D.-I. Zhu W. L. Shin S. Y. Hahm K.-S. Kim Y. (2007). Solution structures and biological functions of the antimicrobial peptide, arenicin-1, and its linear derivative. *Biopolymers* 88 208–216. 10.1002/bip.20700 17285588

[B23] Lee J. Y. Yang S.-T. Lee S. K. Jung H. H. Shin S. Y. Hahm K.-S. (2008). Salt-resistant homodimeric bactenecin, a cathelicidin-derived antimicrobial peptide. *FEBS J.* 275 3911–3920. 10.1111/j.1742-4658.2008.06536.x 18616463

[B24] Mansour S. C. Fuente-Núñez C. Hancock R. E. W. (2015). Peptide IDR-1018: modulating the immune system and targeting bacterial biofilms to treat antibiotic-resistant bacterial infections. *J. Peptide Sci.* 21 323–329. 10.1002/psc.2708 25358509

[B25] McDonald S. K. Fleming K. G. (2016). Aromatic side chain water-to-lipid transfer free energies show a depth dependence across the membrane normal. *J. Am. Chem. Soc.* 138 7946–7950. 10.1021/jacs.6b03460 27254476 PMC4927395

[B26] Mueller P. Rudin D. O. Tien H. T. Wescott W. C. (1962). Reconstitution of cell membrane structure in vitro and its transformation into an excitable system. *Nature* 194 979–980. 10.1038/194979a0 14476933

[B27] Myshakina N. S. Ahmed Z. Asher S. A. (2008). Dependence of amide vibrations on hydrogen bonding. *J. Phys. Chem. B* 112 11873–11877. 10.1021/jp8057355 18754632 PMC2633779

[B28] Nagaoka I. Hirota S. Yomogida S. Ohwada A. Hirata M. (2000). Synergistic actions of antibacterial neutrophil defensins and cathelicidins. *Inflamm. Res.* 49 73–79. 10.1007/s000110050561 10738945

[B29] Panteleev P. V. Balandin S. V. Ivanov V. T. Ovchinnikova T. V. (2017). A therapeutic potential of animal β-hairpin antimicrobial peptides. *Curr. Med. Chem.* 24 1724–1746. 10.2174/0929867324666170424124416 28440185

[B30] Panteleev P. V. Bolosov I. A. Kalashnikov A. A. Kokryakov V. N. Shamova O. V. Emelianova A. A. (2018). Combined antibacterial effects of goat cathelicidins with different mechanisms of action. *Front. Microbiol.* 9:2983. 10.3389/fmicb.2018.02983 30555455 PMC6284057

[B31] Panteleev P. V. Bolosov I. A. Ovchinnikova T. V. (2016). Bioengineering and functional characterization of arenicin shortened analogs with enhanced antibacterial activity and cell selectivity. *J. Pept. Sci.* 22 82–91. 10.1002/psc.2843 26814379

[B32] Panteleev P. V. Ovchinnikova T. V. (2017). Improved strategy for recombinant production and purification of antimicrobial peptide tachyplesin I and its analogs with high cell selectivity. *Biotechnol. Appl. Biochem.* 64 35–42. 10.1002/bab.1456 26549611

[B33] Parachin N. S. Mulder K. C. Viana A. A. B. Dias S. C. Franco O. L. (2012). Expression systems for heterologous production of antimicrobial peptides. *Peptides* 38 446–456. 10.1016/j.peptides.2012.09.020 23022589

[B34] Porcelli F. Buck-Koehntop B. A. Thennarasu S. Ramamoorthy A. Veglia G. (2006). Structures of the dimeric and monomeric variants of magainin antimicrobial peptides (MSI-78 and MSI-594) in micelles and bilayers, determined by NMR spectroscopy. *Biochemistry* 45 5793–5799. 10.1021/bi0601813 16669623

[B35] Pyrkov T. V. Chugunov A. O. Krylov N. A. Nolde D. E. Efremov R. G. (2009). PLATINUM: a web tool for analysis of hydrophobic/hydrophilic organization of biomolecular complexes. *Bioinformatics* 25 1201–1202. 10.1093/bioinformatics/btp111 19244385

[B36] Raj P. A. Karunakaran T. Sukumaran D. K. (2000). Synthesis, microbicidal activity, and solution structure of the dodecapeptide from bovine neutrophils. *Biopolymers* 53 281–292. 10.1002/(SICI)1097-0282(20000405)53:4<281::AID-BIP1<3.0.CO;2-2 10685049

[B37] Romeo D. Skerlavaj B. Bolognesi M. Gennaro R. (1988). Structure and bactericidal activity of an antibiotic dodecapeptide purified from bovine neutrophils. *J. Biol. Chem.* 263 9573–9575. 3290210

[B38] Rule G. S. Hitchens T. K. (2006). *Fundamentals of Protein NMR Spectroscopy.* Dordrecht: Springer Netherlands, 532.

[B39] Saravanan R. Bhattacharjya S. (2011). Oligomeric structure of a cathelicidin antimicrobial peptide in dodecylphosphocholine micelle determined by NMR spectroscopy. *Biochim. Biophys. Acta* 1808 369–381. 10.1016/j.bbamem.2010.10.001 20933496

[B40] Schilling S. Wasternack C. Demuth H.-U. (2008). Glutaminyl cyclases from animals and plants: a case of functionally convergent protein evolution. *Biol. Chem.* 389 983–991. 10.1515/BC.2008.111 18979624

[B41] Schmidt E. Güntert P. (2015). Automated structure determination from NMR spectra. *Methods Mol. Biol.* 1261 303–329. 10.1007/978-1-4939-2230-7_16 25502206

[B42] Selsted M. E. (2004). Theta-defensins: cyclic antimicrobial peptides produced by binary ligation of truncated alpha-defensins. *Curr. Protein Pept. Sci.* 5 365–371. 10.2174/1389203043379459 15544531

[B43] Shamova O. Orlov D. Stegemann C. Czihal P. Hoffmann R. Brogden K. (2009). ChBac3.4: a novel proline-rich antimicrobial peptide from goat leukocytes. *Int. J. Pept. Res. Ther.* 15 31–42. 10.1007/s10989-008-9159-7

[B44] Shenkarev Z. O. Balandin S. V. Trunov K. I. Paramonov A. S. Sukhanov S. V. Barsukov L. I. (2011). Molecular mechanism of action of β-hairpin antimicrobial peptide arenicin: oligomeric structure in dodecylphosphocholine micelles and pore formation in planar lipid bilayers. *Biochemistry* 50 6255–6265. 10.1021/bi200746t 21627330

[B45] Stavrakoudis A. Tsoulos I. G. Shenkarev Z. O. Ovchinnikova T. V. (2009). Molecular dynamics simulation of antimicrobial peptide arenicin-2: beta-hairpin stabilization by noncovalent interactions. *Biopolymers* 92 143–155. 10.1002/bip.21149 19189382

[B46] Storici P. Tossi A. Lenarčič B. Romeo D. (1996). Purification and structural characterization of bovine cathelicidins, precursors of antimicrobial peptides. *Eur. J. Biochem.* 238 769–776. 10.1111/j.1432-1033.1996.0769w.x 8706679

[B47] Sychev S. V. Balandin S. V. Panteleev P. V. Barsukov L. I. Ovchinnikova T. V. (2015). Lipid-dependent pore formation by antimicrobial peptides arenicin-2 and melittin demonstrated by their proton transfer activity. *J. Pept. Sci.* 21 71–76. 10.1002/psc.2724 25522354

[B48] Sychev S. V. Panteleev P. V. Ovchinnikova T. V. (2017a). Structural study of the β-hairpin marine antimicrobial peptide arenicin-2 in PC/PG lipid bilayers by fourier transform infrared spectroscopy. *Russ. J Bioorg. Chem.* 43 502–508. 10.1134/S1068162017050144

[B49] Sychev S. V. Sukhanov S. V. Panteleev P. V. Shenkarev Z. O. Ovchinnikova T. V. (2017b). Marine antimicrobial peptide arenicin adopts a monomeric twisted β-hairpin structure and forms low conductivity pores in zwitterionic lipid bilayers. *Biopolymers* 110:e23093. 10.1002/bip.23093 29266227

[B50] Usachev K. S. Efimov S. V. Kolosova O. A. Klochkova E. A. Aganov A. V. Klochkov V. V. (2015). Antimicrobial peptide protegrin-3 adopt an antiparallel dimer in the presence of DPC micelles: a high-resolution NMR study. *J. Biomol. NMR*. 62 71–79. 10.1007/s10858-015-9920-0 25786621

[B51] Whelehan C. J. Barry-Reidy A. Meade K. G. Eckersall P. D. Chapwanya A. Narciandi F. (2014). Characterisation and expression profile of the bovine cathelicidin gene repertoire in mammary tissue. *BMC Genomics* 15:128. 10.1186/1471-2164-15-128 24524771 PMC3932039

[B52] Wiegand I. Hilpert K. Hancock R. E. W. (2008). Agar and broth dilution methods to determine the minimal inhibitory concentration (MIC) of antimicrobial substances. *Nat. Protoc.* 3 163–175. 10.1038/nprot.2007.521 18274517

[B53] Wu M. Maier E. Benz R. Hancock R. E. (1999). Mechanism of interaction of different classes of cationic antimicrobial peptides with planar bilayers and with the cytoplasmic membrane of *Escherichia coli*. *Biochemistry* 38 7235–7242. 10.1021/bi9826299 10353835

[B54] Yang D. Kay L. E. (1996). Contributions to conformational entropy arising from bond vector fluctuations measured from NMR-derived order parameters: application to protein folding. *J. Mol. Biol.* 263 369–382. 10.1006/jmbi.1996.0581 8913313

[B55] Yonezawa A. Kuwahara J. Fujii N. Sugiura Y. (1992). Binding of tachyplesin I to DNA revealed by footprinting analysis: significant contribution of secondary structure to DNA binding and implication for biological action. *Biochemistry* 31 2998–3004. 10.1021/bi00126a022 1372516

[B56] Zerweck J. Strandberg E. Kukharenko O. Reichert J. Bürck J. Wadhwani P. (2017). Molecular mechanism of synergy between the antimicrobial peptides PGLa and magainin 2. *Sci. Rep.* 7:13153. 10.1038/s41598-017-12599-7 29030606 PMC5640672

[B57] Zhang G.-W. Lai S.-J. Yoshimura Y. Isobe N. (2014). Expression of cathelicidins mRNA in the goat mammary gland and effect of the intramammary infusion of lipopolysaccharide on milk cathelicidin-2 concentration. *Vet. Microbiol.* 170 125–134. 10.1016/j.vetmic.2014.01.029 24572177

