# Supplementary material for: Dodecapeptide Cathelicidins of Cetartiodactyla: Structure, Mechanism of Antimicrobial Action, and Synergistic Interaction With Other Cathelicidins
Source: Front Microbiol. 2021 Aug 13;12:725526. doi: 10.3389/fmicb.2021.725526 (PMC8415029; doi:10.3389/fmicb.2021.725526)
Supplement: Supplementary file 1 [file Data_Sheet_1.docx]

Supplementary Material

Ilia A. Bolosov^1^, Pavel V. Panteleev^1,2^, Sergei V. Sychev^1^, Stanislav V. Sukhanov^1^, Pavel A. Mironov^1,3^, Mikhail Yu. Myshkin^1^, Zakhar O. Shenkarev^1,2^, Tatiana V. Ovchinnikova^1,2,3*^

^1^M.M. Shemyakin & Yu.A. Ovchinnikov Institute of Bioorganic Chemistry, the Russian Academy of Sciences, Moscow 117997, Russia;

^2^Phystech School of Biological and Medical Physics, Moscow Institute of Physics and Technology (State University), 141701 Dolgoprudny, Moscow Region, Russia

^3^ Faculty of Biology, Lomonosov Moscow State University, 119234 Moscow, Russia

*** Correspondence:**Tatiana V. Ovchinnikova

ovch@ibch.ru;

ovch@bk.ru

# Supplementary Material and Methods

## Polyacrylamide gel electrophoresis

The expression level of the recombinant proteins was monitored using sodium dodecyl sulphate polyacrylamide gel electrophoresis (SDS-PAGE) according to the basic Laemmli procedure (Laemmli, 1970) in 15% separating gel. Recombinant peptides were analyzed by Tricine-SDS-PAGE in 16.5% gel containing 6 M urea (Schägger, 2006). All the samples were mixed with 100 mM Tris/HCl buffer (pH 6.8) containing 1% SDS, 8 M urea, 0.01% Coomassie G-250 in the presence or the absence of 1% 2-mercaptoethanol prior and applied to SDS-PAGE. The gels were ﬁxed and stained in 0.025% Coomassie G-250 solution containing 50% methanol and 10% acetic acid, and then washed with 5% acetic acid.

## MALDI-TOF MS

The analysis was performed using MALDI-TOF mass spectrometry at the “Human proteome” Core Facility at the Department of proteomic research (IBMC). MALDI-TOF mass spectrometry studies were performed on an Ultraflex instrument (Bruker Daltonics, Bremen, Germany) (Toropygin et al., 2008). Mass spectra were acquired in a positive-ion reflector mode, 256–1500 laser shots were summed per spectrum. As a matrix, we used 2.5-dihydroxybenzoic acid (DHB) in 20% acetonitrile, 0.1% TFA at a concentration of 10 mg/ml. Usually, for the MALDI probe preparation, the dried-droplet method was used: 0.3 ml of 2% TFA was mixed with 0.3 ml of the sample (0.5–2 pmol per target) and 0.3 ml of the matrix solution, then loaded onto a MALDI sample plate, and measured by MS.

## Calculation of bend and twist angles in β-structure

The overall geometry of the ChDode and PcDode β-sheets in the terms of ‘kink’ , ‘twist’ and ‘interdimer cross’ (only for ChDode in DPC) angles was analyzed using coordinates of backbone NH and C’ atoms of the peptides. The ‘kink’ was defined as a cross-angle between two vectors ***a*** and ***b***. For ChDode ***a*** is a mean of the Val6-1:C’–Ile2-1:NH and Ile8-2:C’–Arg12-2:NH vectors, and ***b*** is a mean of the Val6-2:C’–Ile2-2:NH and Ile8-1:C’–Arg12-1:NH vectors (the ‘-1’ and ‘-2’ suffixes denote the first and second peptide chains in the ChDode dimer or in the tetramer). For PcDode we chose a mean of the I6:C`-C3:NH and C23:C`-I20:NH as vector ***a*** and C11:C`-V8:NH and T18:C`-C15:NH as vector ***b***. The ‘twist’ was defined as a cross-angle between projection of two vectors for ChDode Cys3-1:NH-Cys11-2:C’ and Cys11-1:NH-Cys3-2:C’ to the plane perpendicular to long axis of the β-sheet (mean of the Cys3-1:NH-Cys11-1:NH and Cys11-2:C’-Cys3-3:C’ vectors). ‘Twist’ angle for PcDode molecule was calculated using vectors Cys3:NH-Cys11:C` and Cys15:NH-Cys23:C`. The ‘interdimer cross’ angle for ChDode in DPC was defined as a cross-angle between projection of two vectors ***a*** and ***b***, where ***a*** is a mean of the Cys3-1:C’-Cys11-1:C’ and Cys11-2:C’-Cys3-2:C’ vectors, and ***b*** is a mean of the Cys3-3:C’-Cys11-3:C’ and Cys11-4:C’-Cys3-4:C’ vectors, to the plane perpendicular to the vector ***c***, connecting the centers of two covalent dimers (coordinate of each center was defined as the mean coordinate of C’ atoms of all four cysteine residues). (The ‘-3’ and ‘-4’ suffixes denote the third and fourth peptide chains in the ChDode tetramer.)

# Supplementary References

Laemmli, U. K. (1970). Cleavage of structural proteins during the assembly of the head of bacteriophage T4. *Nature* 227, 680–685. doi:10.1038/227680a0.

Schägger, H. (2006). Tricine-SDS-PAGE. *Nat. Protoc.* 1, 16–22. doi:10.1038/nprot.2006.4.

Toropygin, I. Y., Kugaevskaya, E. V., Mirgorodskaya, O. A., Elisseeva, Y. E., Kozmin, Y. P., Popov, I. A., et al. (2008). The N-domain of angiotensin-converting enzyme specifically hydrolyzes the Arg-5-His-6 bond of Alzheimer’s Abeta-(1-16) peptide and its isoAsp-7 analogue with different efficiency as evidenced by quantitative matrix-assisted laser desorption/ionization time-of-flight mass spectrometry. *Rapid Commun. Mass Spectrom. RCM* 22, 231–239. doi:10.1002/rcm.3357.

**Supplementary Table S1.** Amino acid sequences of natural dodecapeptide cathelicidins (CATHL1)

| Animal | Family | Sequence | GenBank ID |
| --- | --- | --- | --- |
| *Sousa chinensis* | Delphinidae | QRCRIIVIRMCP | QWLN01032320.1, RWJT01022817.1 |
| *Globicephala melas* | Delphinidae | QRCRIIVIRMCP | SWEB01014618.1 |
| *Tursiops aduncus* | Delphinidae | QRCRIIVIRMCR | NCQN01000307.1 |
| *Tursiops truncatus* | Delphinidae | QRCRIIVIRMCR | QUXD02031530.1, QMGA01000085.1 |
| *Orcinus orca* | Delphinidae | QRCRIIVIRTCR | ANOL02069867.1 |
| *Physeter catodon* | Physeteridae | QICRIIVVRVCRPICRITVIRVCS | UEMC01000008.1, PGGR01038967.1, AWZP01092890.1 |
| *Lipotes vexillifer* | Lipotidae | QRYRIIVIRVCR | AUPI01005484.1 |
| *Pontoporia blainvillei* | Pontoporiidae | QRCRRIVIRLCP | RJWI010009523.1 |
| *Mesoplodon bidens* | Ziphiidae | QVCRIIVMRMCR | PVJJ010007056.1 |
| *Ziphius cavirostris* | Ziphiidae | QMCRIVVIRTCR | RJWS010169603.1 |
| *Eubalaena japonica* | Balaenidae | RICRVIVMRVCR | RJWP010071972.1 |
| *Moschus chrysogaster* | Moschidae | RLCHIVVIRVCR | SPDY01014653.1 |
| *Moschus berezovskii* | Moschidae | RLCHIVVIRVCR | SPDX01004240.1, SPDX01009396.1, SGQJ01001806.1 |
| *Moschus moschiferus* | Moschidae | RLCHIVVIRVCR | PVHU010018228.1 |
| *Giraffa tippelskirchi* | Giraffidae | RLCRIIVMRICR | LVKQ01077977.1 |
| *Okapia johnstoni* | Giraffidae | RICRIIVMRICR | LVCL010151530.1 |
| *Antilocapra americana* | Antilocapridae | RLCRIVVIRICR | PVKT010030555.1 |
| *Tragulus javanicus* | Tragulidae | QVCRIVVLRVCR | PVHZ010007883.1 |
| *Tragulus kanchil* | Tragulidae | QVCRIVVLRVCR | SJXW01019505.1 |
| *Odocoileus hemionus* | Cervidae | RVCRIVVIRVCR | RFLZ01135669.1 |
| *Elaphurus davidianus* | Cervidae | RVCRIVVIRVCR | JRFZ01141180.1, JRFZ01035751.1 |
| *Odocoileus virginianus* | Cervidae | RVCRIVVIRVCR | MLBE01000123.1 |
| *Rangifer tarandus* | Cervidae | RVCRIVVIRVCR | PVIN010072712.1, PVIN010085571.1 |
| *Przewalskium albirostris* | Cervidae | RVCRIVVIRVCR | SJXR01031456.1 |
| *Przewalskium albirostris* | Cervidae | RVCRIVVLRVCR | SJXR01001537.1 |
| *Muntiacus muntjak* | Cervidae | RVCRIVVLRVCR | SJXU01045938.1 |
| *Muntiacus crinifrons* | Cervidae | RVCRIVVLRVCR | SJXS01011712.1, SJXS01015479.1 |
| *Muntiacus reevesi* | Cervidae | *VCRIVVLRVCR | SJXT01015098.1 |
| *Muntiacus crinifrons* | Cervidae | RVCCIVVLRVCR | SJXS01015479.1 |
| *Muntiacus reevesi* | Cervidae | RVCHFVVIRVCR | SJXT01015098.1 |
| *Muntiacus muntjak* | Cervidae | RVCRVVMLSVCR | SJXU01045938.1 |
| *Muntiacus crinifrons* | Cervidae | RVCHFVVLRVCR | SJXS01011712.1 |
| *Axis porcinus* | Cervidae | RVCRFIVIRICR | QQTR01119330.1 |
| *Axis porcinus* | Cervidae | RVCRFIVIRVCR | QQTR01119330.1 |
| *Pantholops hodgsonii* | Bovidae | RLCRIVVIRVCR | AGTT01128678.1 |
| *Bos mutus* | Bovidae | RLCRIVVIRVCR | AGSK01178572.1 |
| *Procapra przewalskii* | Bovidae | RLCRIVVIRVCR | SJYF01000158.1 |
| *Bos grunniens* | Bovidae | RLCRIVVIRVCR | VBZB01000023.1 |
| *Ovis aries* | Bovidae | RICRIIFLRVCR | AMGL02030114.1, PEKD01003985.1, CBYI010014275.1, PEKD01003984.1 |
| *Ovis ammon* | Bovidae | RICRIIFLRVCR | NIWH01002802.1, SJYP01000045.1 |
| *Hemitragus hylocrius* | Bovidae | RICRIIFLRVCR | PVJR01013055.1 |
| *Capra hircus* | Bovidae | RICQFVLIRVCR | GFGD01030961.1, GAOD01015421.1, AJPT02103479.1, LWLT01000024.1 |
| *Capra sibirica* | Bovidae | RICQFVLIRVCR | NIYN02061807.1 |
| *Capra aegagrus* | Bovidae | RICQFVLIRVCR | JXYW01197179.1, CBYH010001764.1 |
| *Ammotragus lervia* | Bovidae | RICQFVLIRVCR | NIVO01006316.1 |
| *Ovis canadensis* | Bovidae | RICHFVLIRVCR | PVIS010069390.1 |
| *Redunca redunca* | Bovidae | RTCRFIVIRVCR | SJYQ01012660.1 |
| *Neotragus moschatus* | Bovidae | RTCRFIVIRVCR | SJYA01002699.1 |
| *Nanger granti* | Bovidae | RIGRIIVIRVCR | SJYD01061367.1 |
| *Eudorcas thomsonii* | Bovidae | RIGRIIVIRVCR | SJYE01122334.1 |
| *Antidorcas marsupialis* | Bovidae | QIGCIVVIRVCR | SJYB01007930.1 |
| *Litocranius walleri* | Bovidae | QIGCIVVIRVCR | SJYG01000096.1 |
| *Cephalophus harveyi* | Bovidae | RICRIVVIRVCR | SJYT01004901.1 |
| *Neotragus pygmaeus* | Bovidae | RICRIVVIRVCR | SJYV01030841.1 |
| *Hippotragus niger* | Bovidae | RICRIVVIRVCR | VHQK01014889.1 |
| *Hydropotes inermis* | Bovidae | RICRIVVIRVCR | SPDZ01001416.1 |
| *Oreotragus oreotragus* | Bovidae | RICRIVVIRVCR | SJYX01017676.1 |
| *Saiga tatarica* | Bovidae | RICRIVVIRVCR | PVIK010200420.1 |
| *Tragelaphus strepsiceros* | Bovidae | RSCRFVIIRICR | SJYU01005166.1 |
| *Tragelaphus oryx* | Bovidae | RSCRFVIIRICR | SJYK013935403.1 |
| *Tragelaphus spekii* | Bovidae | RSCRFVIIRICR | SJYL010054201.1 |
| *Tragelaphus eurycerus* | Bovidae | RSCRFVIIRICR | SJYI010231274.1 |
| *Tragelaphus scriptus* | Bovidae | RSCRFVIIRICR | SJXY01000211.1 |
| *Tragelaphus buxtoni* | Bovidae | RSCRFVIIRICR | SJYJ010341703.1 |
| *Syncerus caffer* | Bovidae | *LCRIVVIRVCR | SJXX01000493.1 |
| *Oryx gazella* | Bovidae | RICRIMVIRVCR | RAWW01009131.1 |
| *Pseudois nayaur* | Bovidae | RICRFVFIRVCR | NIZD01012642.1 |
| *Bubalus bubalis* | Bovidae | RFCRFVVIRVCR | AWWX01503677.1 |
| *Bubalus bubalis* | Bovidae | RLCRYIVIRVCR | ACZF03004309.1, PZYV01000014.1 |
| *Beatragus hunteri* | Bovidae | RMCRYIIIRVCR | PVKQ01021889.1 |
| *Kobus ellipsiprymnus* | Bovidae | RTCRFIVIRICR | SJYR01003864.1 |
| *Damaliscus lunatus* | Bovidae | RMCRYIIIRVCR | SJXP01002955.1 |
| *Raphicerus campestris* | Bovidae | RICRIVVLRVCR | SJYW01008136.1 |
| *Sylvicapra grimmia* | Bovidae | RTCRIVIIRVCR | SJYH01005526.1 |
| *Connochaetes taurinus* | Bovidae | RMCRIVIIRVCR | SJXO01002629.1 |
| *Aepyceros melampus* | Bovidae | RICRIVVIRMCR | SJXQ01000018.1 |
| *Tragelaphus imberbis* | Bovidae | RSCRFVVIRICR | SJYM01002114.1 |

* - stop codon


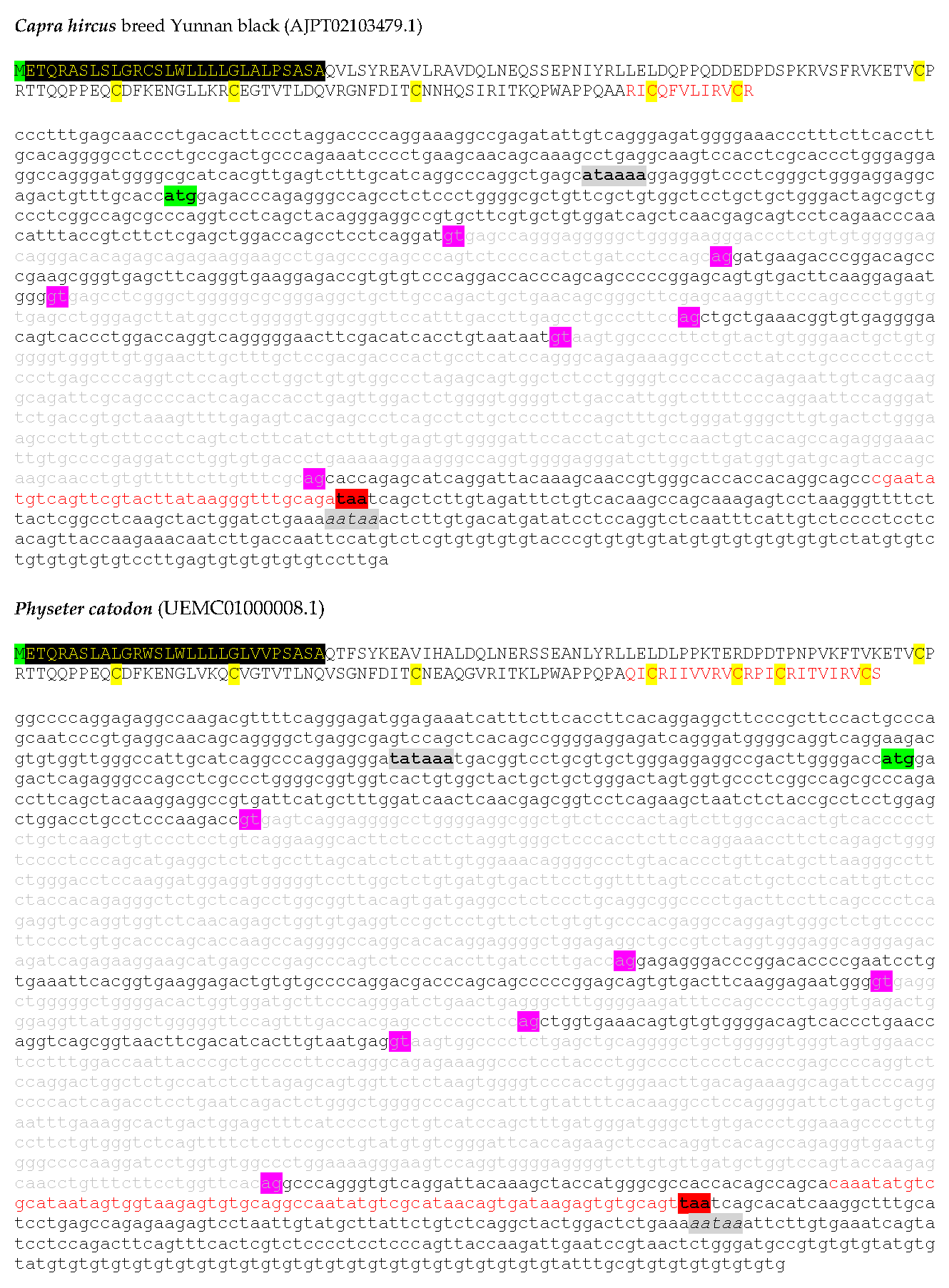


**Figure S1.** Structure of *CATHL1* genes and corresponding preprocathelicidins of *Capra hircus* and *Physeter catodon*. Predicted intron/exon boundaries are highlighted in violet. Signal peptide sequences are marked in yellow/black. Nucleotide and peptide sequences of putative mature cathelicidins are marked with red font. The TATA-box signal is in bold and grey marked and the polyadenylation signal is in italics and grey marked.


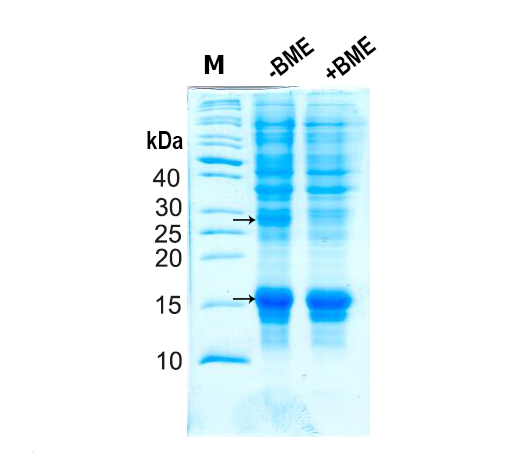


**Figure S2.** SDS-PAGE of the total lysate of *Escherichia coli* BL21 (DE3) cells after IPTG induction with or without β-mercaptoethanol (BME). M – molecular mass standard. The arrows point at the target fusion protein in the monomeric (14.7 kDa) or dimeric (29.3 kDa) form.


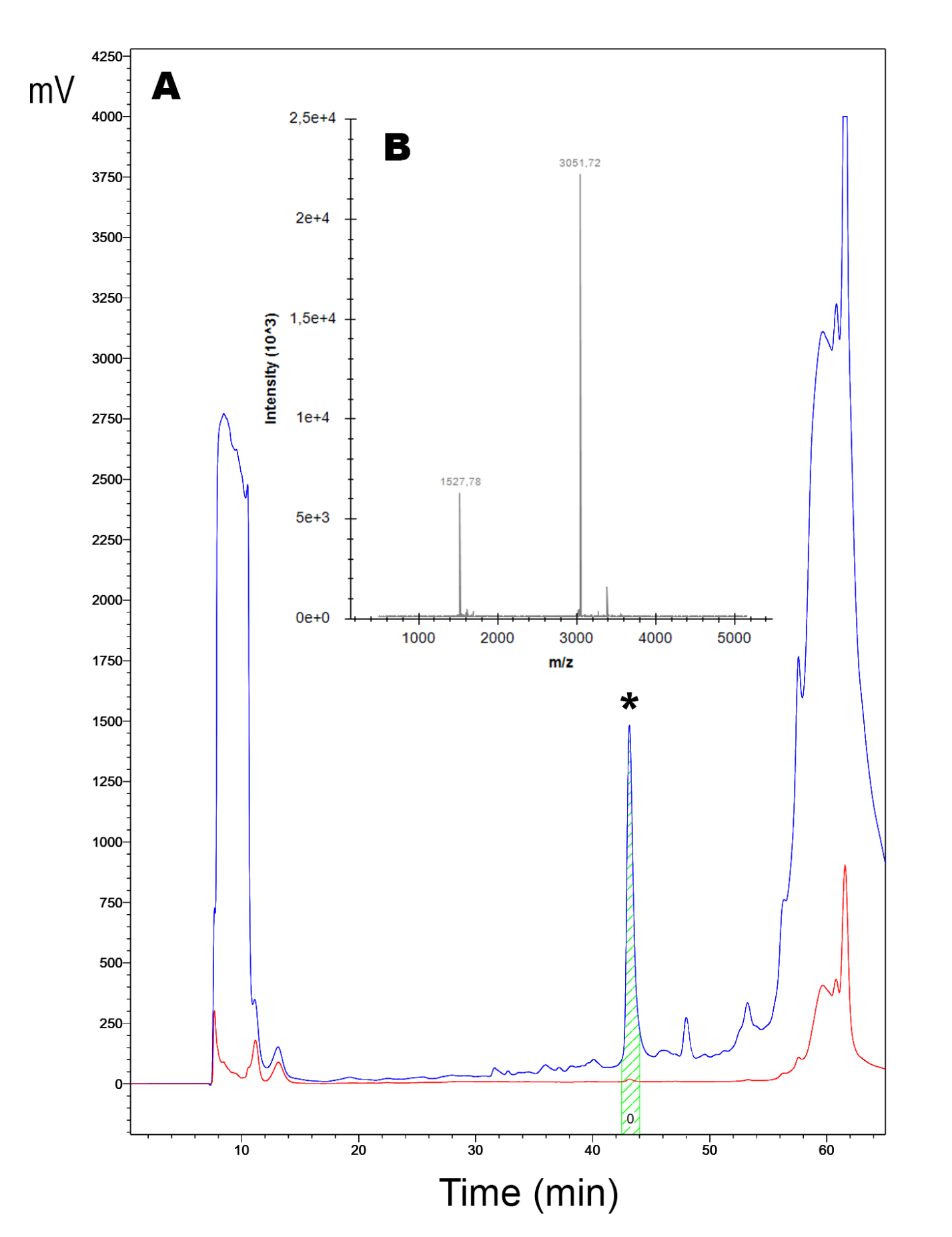


**Figure S3.** **(A)** Reverse-phase high-performance liquid chromatography (RP-HPLC) purification of the recombinant ^15^N-ChDode, was performed with a linear gradient of acetonitrile (0 – 80% for 65 min) in water containing 0.1% trifluoroacetic acid with flow rate of 2 ml/min. The peaks were monitored at 214 (blue line) and 280 nm (red line). The fraction of the target peptide is marked with an asterisk. **(B)** MALDI-TOF mass spectrometry analysis of the recombinant ^15^N-ChDode. The experimental [M+H]^+^ monoisotopic masses are presented in the picture. Mass spectra were acquired in a positive-ion reflector mode, 256–1500 laser shots were summed per spectrum.


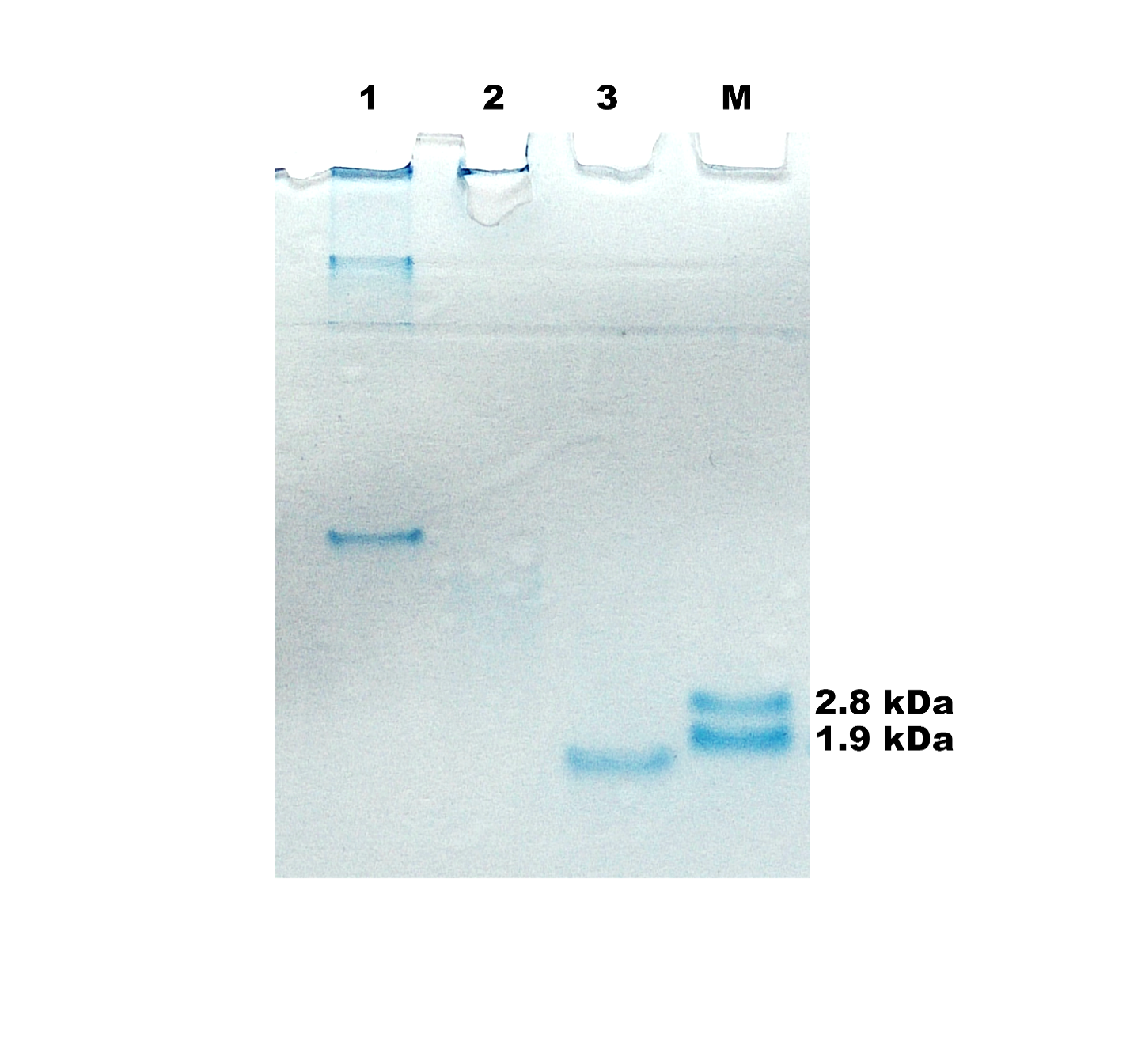


**Figure S4.** Tricine-SDS-polyacrylamide gel electrophoresis (Tricine-SDS-PAGE) of the recombinant ChDode: 1— the recombinant ChDode (0.5 µg); 2— the boiled recombinant ChDode (0.5 µg); 3—the recombinant ChDode (0.5 µg) reduced with β-mercaptoethanol; M—molecular mass marker (antimicrobial peptides tritrpticin and melittin). For specification of ~~the~~ molecular ~~weight~~ masses of oligomers see Figure 7D.


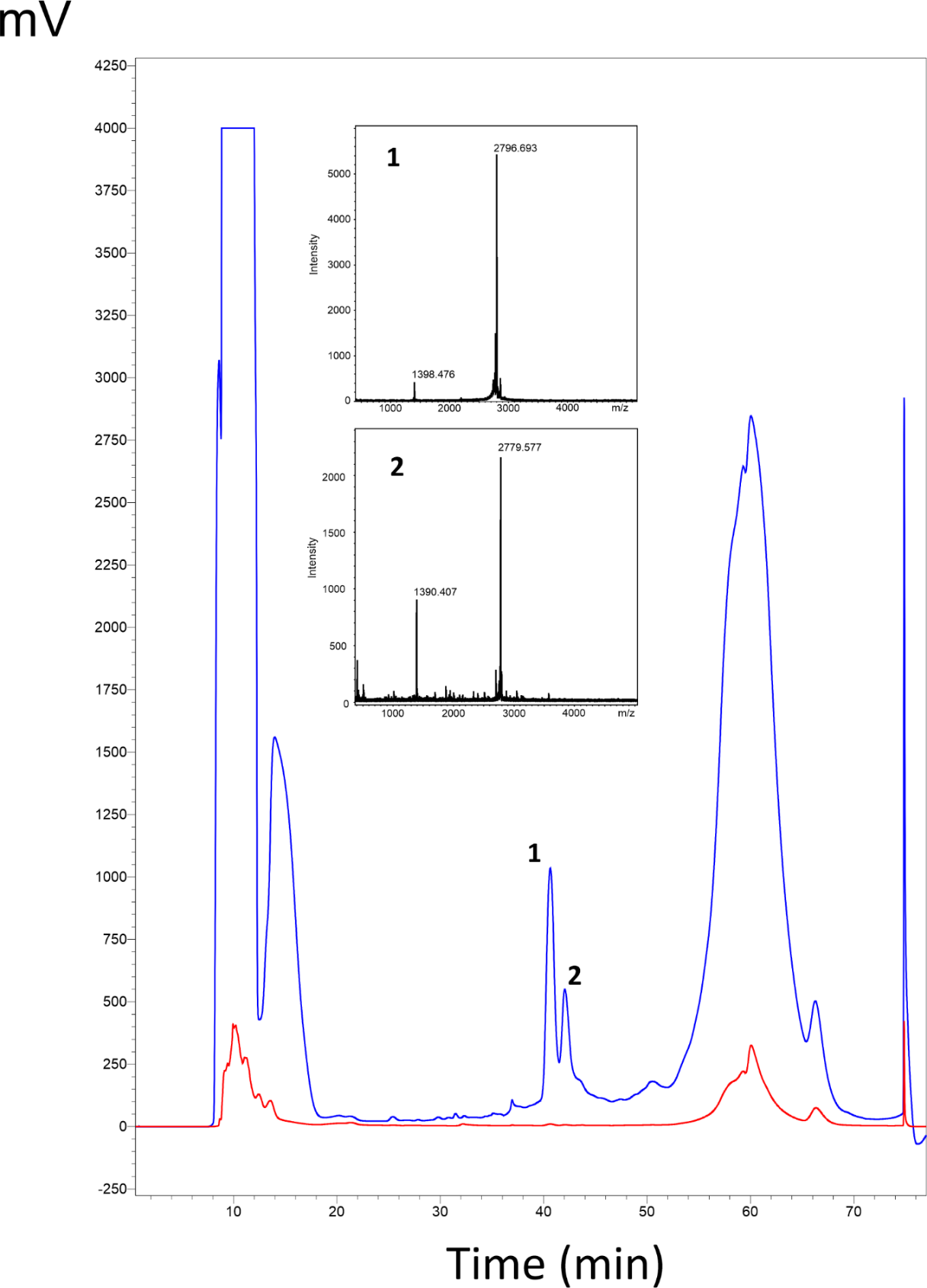


**Figure S5.** Reverse-phase RP-HPLC purification of the recombinant PcDode, was performed with a linear gradient of acetonitrile (0 – 80% for 65 min) in water containing 0.1% trifluoroacetic acid with flow rate of 2 ml/min. The peaks were monitored at 214 (blue line) and 280 nm (red line). Peaks 1 and 2 corresponds to the peptide with the N-terminal glutamine and pyroglutamic acid, respectively. Insert: MALDI-TOF mass spectrometry analysis of the recombinant PcDode fractions 1 and 2. The experimental [M+H]^+^ monoisotopic masses are presented in the picture. Mass spectra were acquired in a positive-ion reflector mode, 256–1500 laser shots were summed per spectrum.

**Supplementary Table S2**. Statistics for the best CYANA structures of ChDode and PcDode in water and DPC micelles.

|  | ChDode H_2_O | ChDode DPC | PcDode H_2_O |
| --- | --- | --- | --- |
| **Distance and angle restraints** |  |  |  |
| Total upper distance restraints (NOE based) | 516 | 569 | 282 |
| Intraresidual | 140 | 298 | 113 |
| Interresidual Intramolecular | 376 | 256 | 169 |
| Sequential (\|*i* – *j*\| = 1) | 118 | 152 | 93 |
| Medium range (1 < \|*i* – *j*\| < 4) | 36 | 12 | 13 |
| Long range (\|*i* – *j*\| > 4) | 222 | 92 | 63 |
| Interresidual Intermolecular (dimer in DPC micelles) |  | 15 |  |
| Unambiguous |  | 13 |  |
| Ambiguous |  | 2 |  |
| Hydrogen bond restraints (bonds/upper/lower) | 12/24/24 | 30/60/60 | 10/20/20 |
| S–S bond restraints (bonds/upper/lower) | 2/6/6 | 4/12/12 | 2/6/6 |
| Torsion angle restraints | 36 | 82 | 39 |
| Angle φ | 22 | 44 | 23 |
| Angle χ_1_ | 14 | 38 | 16 |
| Total restraints/per residue | 612/25.5 | 795/16.6 | 657/27.4 |
| **Statistics for calculated structures** |  |  |  |
| Structures calculated/selected | 20/200 | 20/200 | 20/200 |
| CYANA target function (Å^2^) | 0.26 ± 0.03 | 2.76 ± 0.34 | 1.28± 0.07 |
| Violations of restraints |  |  |  |
| Distance (> 0.2 Å) | 0 | 9 | 1 |
| Distance (> 0.4 Å) | 0 | 0 | 0 |
| Dihedral angles (> 1°) | 0 | 3 | 0 |
| r.m.s.d. (Å) overall |  |  |  |
| Backbone | 0.19 ± 0.05 | 1.11 ± 0.82 | 0.22 ± 0.07 |
| Heavy atoms | 0.98 ± 0.13 | 1.76 ± 0.75 | 0.89 ± 0.12 |

r.m.s.d. – root mean square deviation.

**
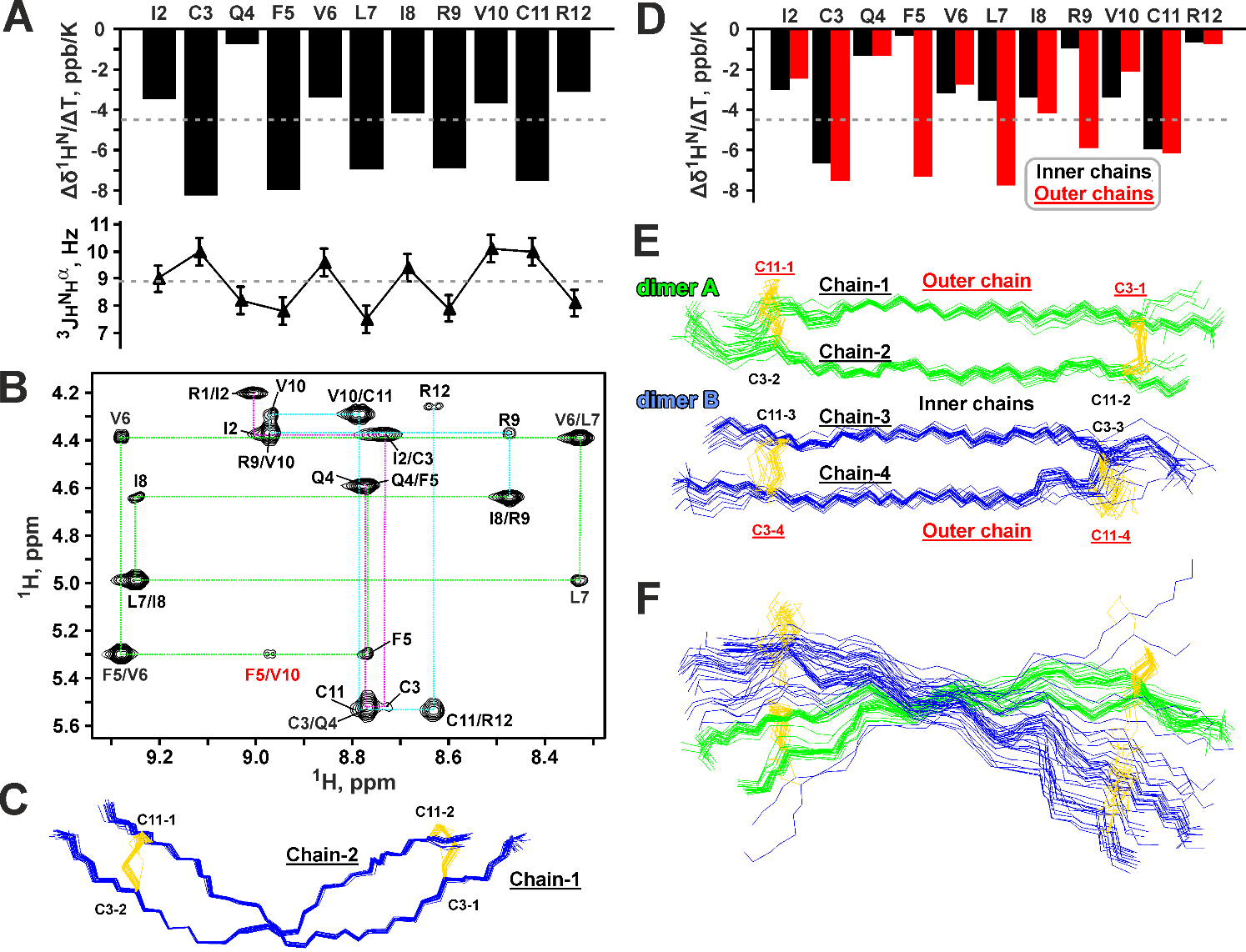
**

**Figure S6.** NMR data defines 3D structure of the ChDode dimer in water (**A-C**) and of the ChDode tetramer in DPC micelles (**D-F**). (**A, D**) Temperature gradients of amide protons (∆δ^1^H^N^/∆T). The values with amplitude <4.5 ppb/K (dashed line) indicated possible participation of HN group in the hydrogen bond formation. The data for the outer chains of the ChDode tetramer in DPC is shown in red (**D**). Panel (**A**) additionally shows ^3^J_H_^N^_H_^α^ values for ChDode in water. Average ^3^J_H_^N^_H_^α^ value (8.9 ± 1.0 Hz) is shown by dashed line. (**B**) HN-Hα region of 2D NOESY (τ_m_ = 100 ms) spectrum of the unlabeled ChDode dimer in water. The colored dotted lines connect intraresidual and sequential cross-peaks showing sequential assignment for the Arg1-Gln4 (magenta), Phe5-Arg9 (green), and Val10-Arg12 (blue) fragments. The intermonomer Phe5:Hα-Val10:HN NOE contact is colored in red. (**C, E, F**) Sets of 20 best structures of the ChDode dimer in water (**C**) and tetramer in DPC micelles (**E, F**). Backbone of the dimer in water are shown in blue. Backbone of the dimers A and B within the tetramer in DPC micelles are shown in green and blue, respectively. Disulfide bonds are in yellow. The structures in panels (**C, E**) are superimposed over all backbone atoms. The structures in panel (**F**) are superimposed over backbone atoms of the dimer A (green) to show various positions of the dimer B (blue).


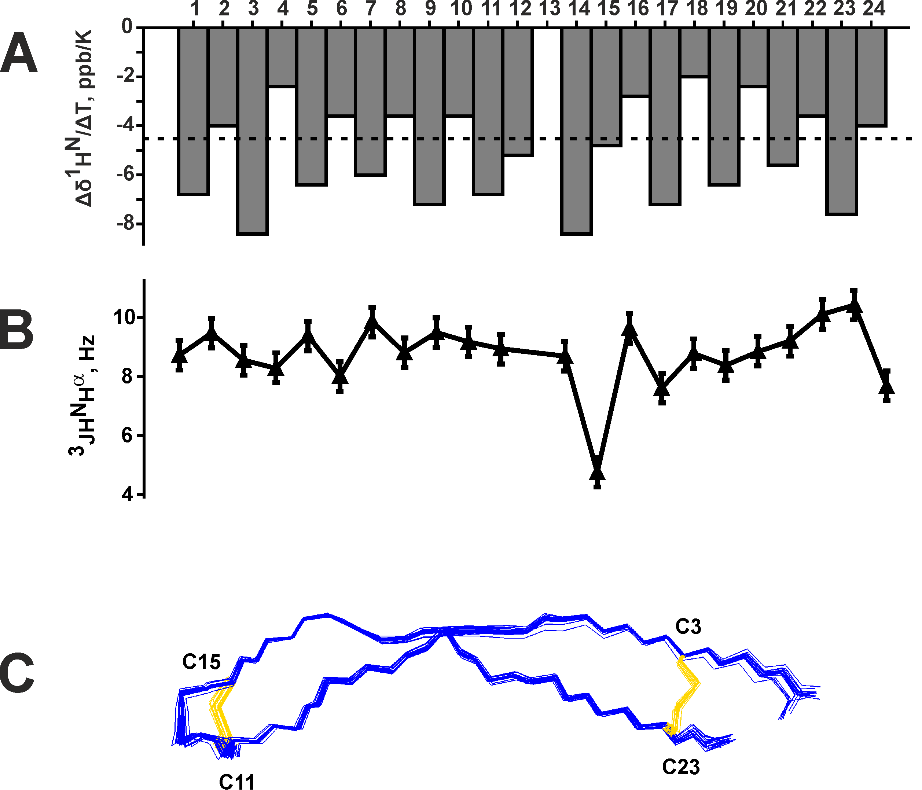


**Figure S7**. NMR data defines 3D structure of the PcDode in water. (**A**) Temperature gradients of amide protons (∆δ^1^H^N^/∆T). The values with amplitude <4.5 ppb/K (dashed line) indicated possible participation of HN group in the hydrogen bond formation. (B) ^3^J_H_^N^_H_^α^ values for ChDode in water. (**C**) Set of 20 best structures of the PcDode in water Disulfide bonds are in yellow. The structures are superimposed over all backbone atoms.


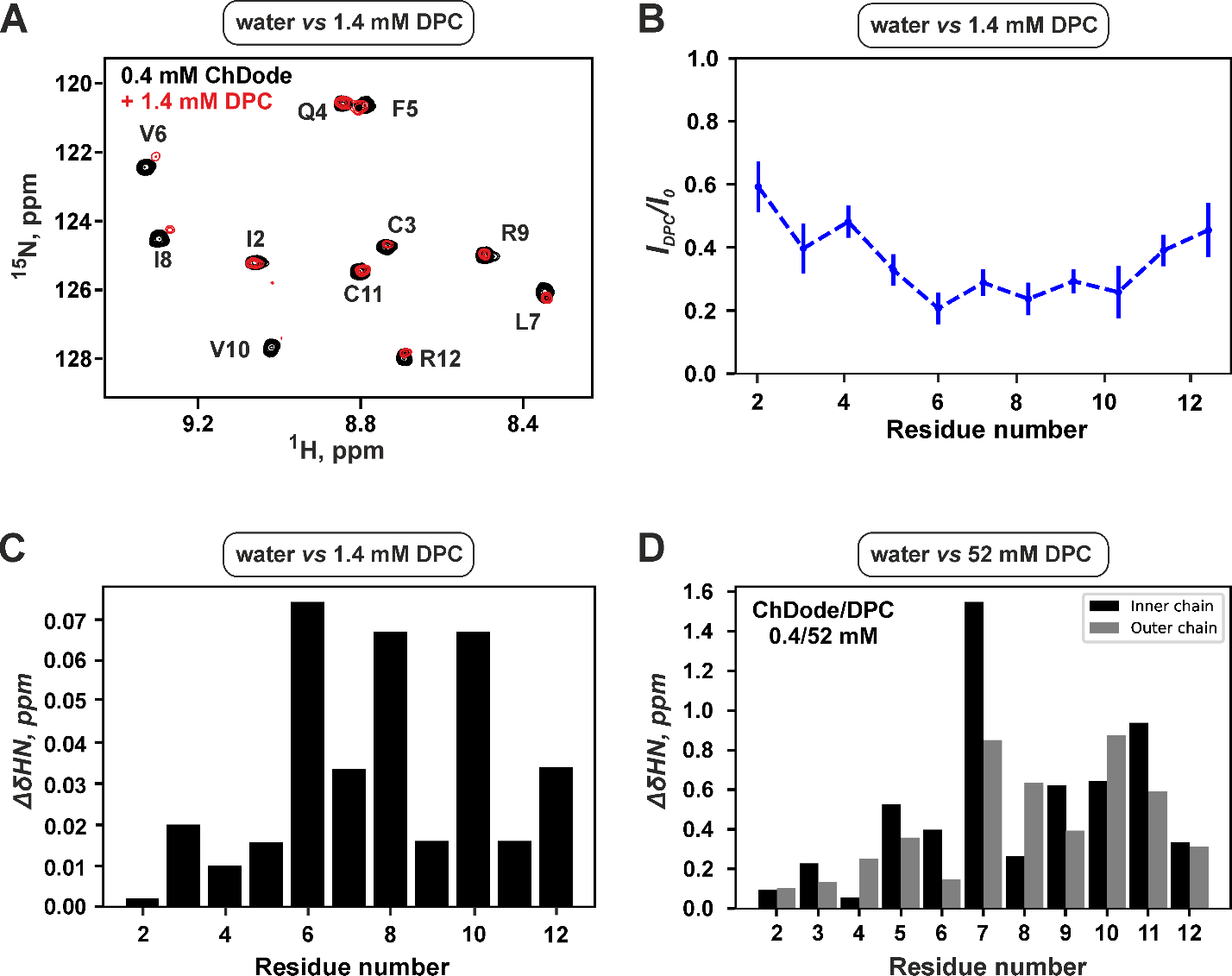


**Figure S8.** Changes of ChDode chemical shifts and signal intensities upon interaction with DPC micelles and dimerization. **(A)** Comparison of 2D ^1^H-^15^N HSQC spectra of 0.4 mM ChDode dimer without (black) and after addition of 1.4 mM of d38-DPC (red, D:L = 1:3.5). (**B,** **C**) Changes in intensities and chemical shifts of ^1^H-^15^N HSQC cross-peaks of ChDode upon addition of 1.4 mM of d38-DPC. (**D**) Differences in ^1^H-^15^N chemical shifts between outer and inner chains of the ChDode tetramer in DPC (D:L = 1:130, 40 °C) and the ChDode dimer in water.


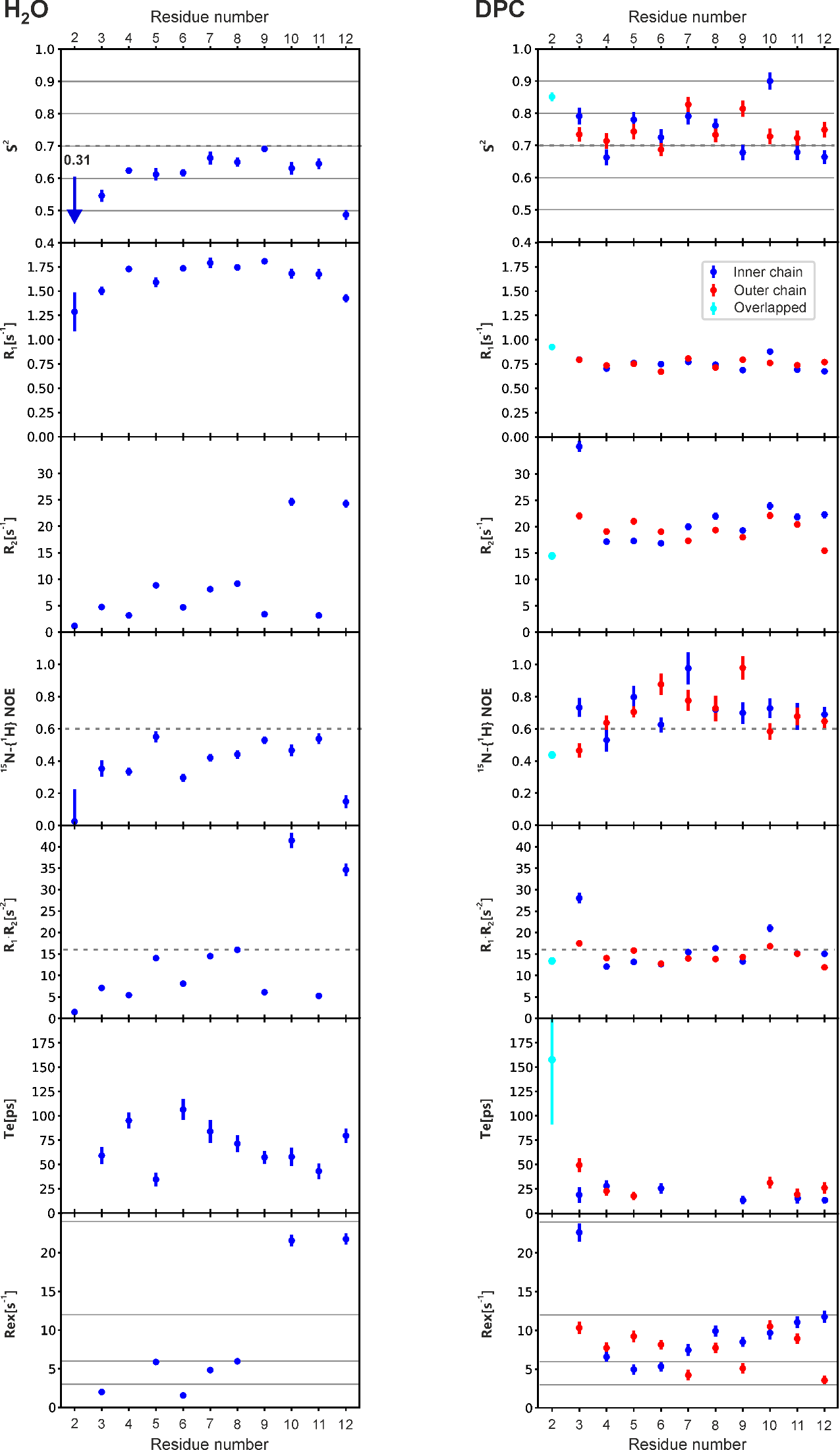


**Figure S9.** ^15^N relaxation data and results of the ‘model-free’ analysis for ChDode dimer in water (left panel, pH 4.0, 30 °C, 800 MHz) and ChDode tetramer in DPC micelles (right panel, pH 4.0, 40 °C, dimer-to-lipid molar ratio (D:L) of 1:130, 800 MHz). In the DPC environment the data for the inner and outer chains of the tetramer are shown in blue and red, respectively. The ^1^H-^15^N signals of Ile2 residue from inner and outer chains were overlapped. The data for Ile2 are shown in cyan. The ^1^H-^15^N signals of first residues are not observable. In both cases, the isotropic overall rotational diffusion model was used. The resulting overall rotational correlation times τ_R_ were 2.1 ns and 9.4 ns for ChDode in water and DPC, respectively. S^2^ – squared values of the generalized order parameter. R_1_ and R_2_ – the values of longitudinal and transverse ^15^N relaxation rates. ^15^N-{^1^H}-NOE - steady-state heteronuclear NOE. τ_e_ – effective correlation times for backbone motions in the ps timescale. R_ex_ – exchange contribution to the transverse relaxation rate. Residues displaying S^2^ < 0.7 and ^15^N-{^1^H}-NOE < 0.6 are subjected to extensive motions in ps-ns timescale. Residues displaying R_1_·R_2_ > 16 s^-2^ [Kneller JM, et al. *J Am Chem Soc*. (2002) 124:1852-3] are subjected to exchange fluctuations in μs-ms timescale.
